# Dynamic matrices with DNA-encoded viscoelasticity for cell and organoid culture

**DOI:** 10.1038/s41565-023-01483-3

**Published:** 2023-08-07

**Authors:** Yu-Hsuan Peng, Syuan-Ku Hsiao, Krishna Gupta, André Ruland, Günter K. Auernhammer, Manfred F. Maitz, Susanne Boye, Johanna Lattner, Claudia Gerri, Alf Honigmann, Carsten Werner, Elisha Krieg

**Affiliations:** 1 Institute for Biofunctional Polymer Materials, Leibniz Institute of Polymer Research Dresden, Dresden, Germany; 2 Center for Regenerative Therapies Dresden, Cluster of Excellence Physics of Life and Faculty of Chemistry and Food Chemistry, Technische Universität Dresden, Dresden, Germany; 3 Institute for Physical Chemistry and Polymer Physics, Polymer Interfaces, Leibniz Institute of Polymer Research Dresden, Dresden, Germany; 4 Institute for Macromolecular Chemistry, Leibniz Institute of Polymer Research Dresden, Dresden, Germany; 5 Max Planck Institute of Molecular Cell Biology and Genetics, Dresden, Germany; 6 Center for Systems Biology Dresden, Dresden, Germany

**Keywords:** Organizing materials with DNA, Molecular self-assembly, Biomaterials, Mechanical properties

## Abstract

Three-dimensional cell and organoid cultures rely on the mechanical support of viscoelastic matrices. However, commonly used matrix materials lack control over key cell-instructive properties. Here we report on fully synthetic hydrogels based on DNA libraries that self-assemble with ultrahigh-molecular-weight polymers, forming a dynamic DNA-crosslinked matrix (DyNAtrix). DyNAtrix enables computationally predictable and systematic control over its viscoelasticity, thermodynamic and kinetic parameters by changing DNA sequence information. Adjustable heat activation allows homogeneous embedding of mammalian cells. Intriguingly, stress-relaxation times can be tuned over four orders of magnitude, recapitulating mechanical characteristics of living tissues. DyNAtrix is self-healing, printable, exhibits high stability, cyto- and haemocompatibility, and controllable degradation. DyNAtrix-based cultures of human mesenchymal stromal cells, pluripotent stem cells, canine kidney cysts and human trophoblast organoids show high viability, proliferation and morphogenesis. DyNAtrix thus represents a programmable and versatile precision matrix for advanced approaches to biomechanics, biophysics and tissue engineering.

## Abstract (web summary)

DNA nanotechnology is used to develop fully synthetic, programmable and printable 3D cell-culture matrices with stress-relaxation crosslinkers that encode (nano)mechanical stability. The hydrogel performs on par with solubilized animal-basement-membrane-derived cell-culture matrices.

## Main

Supramolecular hydrogels are three-dimensional (3D) networks of molecules connected by non-covalent bonds^1,2^. Their dynamic nature gives access to unique properties, such as stimuli responsiveness and self-healing^3,4^. Hydrogels that contain self-assembling DNA are particularly interesting, as the sequence-selective recognition of DNA enables modular construction of complex reconfigurable materials^5–7^. Using principles of DNA nanotechnology, the properties of these systems can be programmed and dynamically changed in situ^8–11^. Among their many applications, DNA-based hydrogels could support 3D cell cultures, for instance, to study mechanisms in developmental biology, recapitulate pathologies and develop (personalized) therapies^12–16^.

The microenvironment of cells in the extracellular matrix is one of the key determinants for cell-fate decisions^17^. Matrix stiffness affects cell proliferation and morphology in 3D organoid cultures via mechanosensitive transcriptional regulators^18,19^. Matrix viscoelasticity entails other critical parameters such as stress relaxation, which describes the time-dependent change of mechanical stress following a deformation^20,21^. Faster stress relaxation can promote cell spreading^22^, migration^23^ and differentiation^24^. However, the most widely used hydrogels for cell culture are animal-derived basement membrane matrices, such as Matrigel; they are poorly tunable, exhibit substantial batch-to-batch variations and can cause unexpected stimulation of cells^25^. More advanced materials that do offer viscoelastic tunability^26–30^ require considerable modifications to the system’s constituents, whose influence on cells is difficult to disentangle from mechanical effects. A DNA-based hydrogel was recently shown to exhibit temperature-dependent stress relaxation^31^. In cell culture, however, temperature must usually remain constant. An ideal cell-instructive substitute material should be chemically defined and adapt its mechanical properties at constant temperature in a programmable manner^17,32^. Moreover, it should be printable under mild cytocompatible conditions to enable top-down fabrication of complex 3D tissue templates^33–35^.

DNA-crosslinked hydrogels appear uniquely suited for addressing the need for a highly adaptive cell-culture platform^36–39^. However, high concentrations of synthetic DNA are typically required, making these materials prohibitively expensive to be deployed at scale. Furthermore, large DNA concentrations can also induce undesired effects when in contact with living cells and tissues. For instance, DNA can electrostatically scavenge positively charged biomolecules^40^ or stimulate inflammation^41,42^. Thus, there has been substantial interest in either reducing the cost of DNA material synthesis^43^ or supporting the network with secondary non-DNA crosslinks^10^.

Here we introduce a dynamic DNA-crosslinked matrix (DyNAtrix), which is based on DNA libraries that self-assemble with a biofunctional polymer. By using programmable crosslinker libraries—rather than simple crosslinker splints—gelation occurs at very low DNA concentrations, and the stiffness of the supramolecular network can be uniquely controlled without changing the DNA concentration or other chemical components. The DNA libraries also provide control over matrix stress relaxation, crosslinking thermodynamics, kinetics and degradability. These features demonstrate the potential of DNA nanotechnology for producing programmable soft materials interacting with living systems.

## Material concept

To create a programmable, cytocompatible and cost-effective material, we synthesized a DNA-functionalized ultrahigh-molecular-weight (UHMW) polymer. We envisioned that this polymer would serve as a blank-slate primary structural scaffold, while small quantities of supplemented DNA-based crosslinkers would modulate the material’s properties (Supplementary Note 2.1). We chose a 3 megadalton poly(acrylamide-co-acrylic acid) backbone due to its anticipated biocompatibility and methanol responsiveness^9,44,45^. The latter allows scalable purification and removal of cytotoxic monomers.

**Fig. 1:**
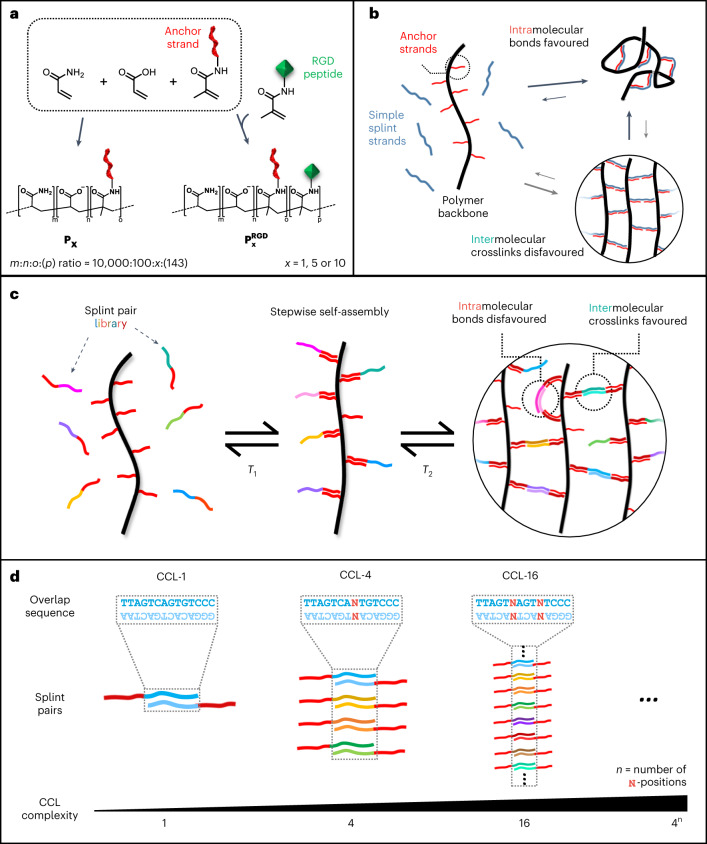
Basic concept and crosslinker design for DyNAtrix. **a**, Polymer synthesis via free radical polymerization of acrylamide, sodium acrylate and acryloyl-based monomers modified with anchor strand DNA (red). For cell culture, acrylamide-labelled cell adhesion peptides with the RGD motif (green) were co-polymerized. **b**, Scheme of intramolecular crosslinks predominant in high-molecular-weight polymers at low concentrations, resulting in low crosslinking efficiency and poor mechanical strength. **c**, Scheme of polymer crosslinking via CCL splints to suppress the formation of intramolecular bonds and promote effective intermolecular crosslinks. **d**, CCLs are generated by introducing ambiguous bases (N) in the overlap domain.

We first synthesized three derivatives, **P**_**1**_, **P**_**5**_ and **P**_**10**_, with average numbers of 3, 20 and 28 covalently attached DNA strands per backbone, respectively (Fig. 1a, Supplementary Figs. 1–3, Supplementary Note 2.2 and Supplementary Table 2). The DNA strands serve as universal anchor sites for non-covalent attachment of DNA-based crosslinker modules. A single polymer batch can thus be used to assemble materials with vastly different properties. For cell-culture studies, synthetic peptides containing the arginine-glycine-aspartate (RGD) motif were linked to the backbone to facilitate cell adhesion and mechanical signalling. The RGD-grafted derivatives, ${{\bf{P}}}_{{\bf{5}}}^{{\bf{R}}{\bf{G}}{\bf{D}}}$ and ${{\bf{P}}}_{{\bf{10}}}^{{\bf{R}}{\bf{G}}{\bf{D}}}$ have similar molecular weights to their peptide-free counterparts (Supplementary Tables 2 and 3).

To our surprise, initial attempts to crosslink the polymers at low concentration with simple splint strands failed to produce stable gels. As gel stiffness is proportional to the number of effective crosslinks (Supplementary Note 2.3), we hypothesized that ineffective intramolecular bonds dominated over network-forming intermolecular crosslinks (Fig. 1b). Intramolecular bonding is not specific to DNA gels, but a general phenomenon in polymer gels, typically reducing crosslink efficiencies to 20% or less^46,47^.

The sequence selectivity of DNA hybridization offered a unique solution to this problem: instead of using a single splint type, we constructed complex libraries based on a dual-splint design (Fig. 1c,d). All library members contain an identical adaptor domain that binds anchor strands below a specific melting temperature, *T*_1_. In addition, each splint contains an overlap domain that pairs with only one other splint below a second melting temperature, *T*_2_. The adaptor and overlap domains were designed to have adequately separated melting temperatures, thus ensuring successive binding when cooled from 95 °C to 20 °C in a one-pot annealing process (Supplementary Fig. 4).

**Fig. 2:**
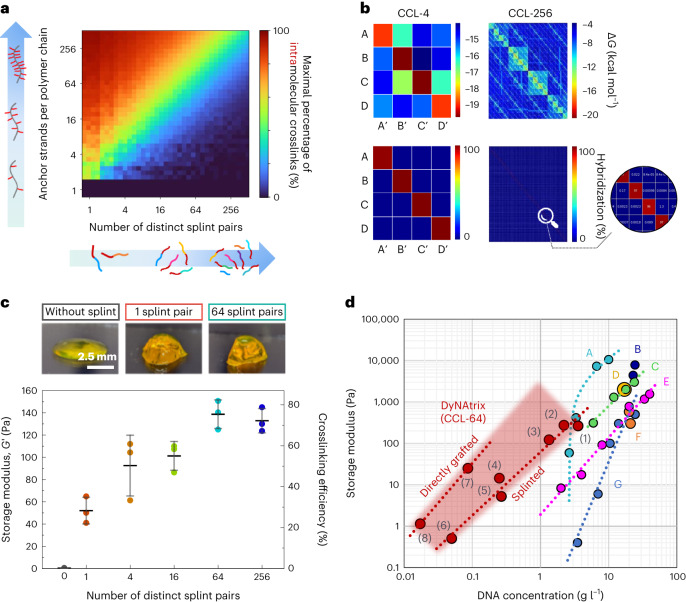
Theoretical prediction and rheological validation of DyNAtrix crosslinked via CCL. **a**, Statistical simulation of the maximum percentage of intramolecular crosslinks as a function of CCL complexity and the number of anchor strands per polymer backbone. **b**, The predicted minimum free energy (top) and the predicted hybridization interactions (normalized Boltzmann distribution; bottom) of crosslinker libraries CCL-4 and CCL-256 at equilibrium. High-resolution matrices for all crosslinker libraries are available on figshare (10.6084/m9.figshare.23309429). **c**, Top: photographs of DyNAtrix, stained with SYBR Gold. Bottom: average stiffness of DyNAtrix (1% (w/v) **P**_**5**_) crosslinked via 1-, 4-, 16-, 64- and 256-splint libraries at 20 °C. The corresponding crosslinking efficiencies were estimated from the affine network model (Supplementary Note 2.3). Crosslink efficiencies of the high-complexity libraries approach the theoretical limit for affine molecular networks. All samples have a total DNA content of 1.3 g l^−1^. Data are shown as mean ± s.d. (*n* = 3). **d**, Different DNA hydrogel designs give access to different concentration and stiffness regimes: comparison between previously reported synthetic DNA-crosslinked hydrogels (A, ref. ^58^; B, ref. ^59^; C, ref. ^60^; D, ref. ^10^; E, ref. ^39^; F, ref. ^61^; G, ref. ^14^) and several DyNAtrix gels (this study, red). DyNAtrix samples (1)–(6) were crosslinked with CCL-64 splints, while samples (7) and (8) had CCL-64 directly grafted onto the polymer backbone (compare Fig. 1c and Supplementary Fig. 10). (1) 1% (w/v) **P**_**10**_; (2) 1.65% (w/v) **P**_**5**_; (3) 1% (w/v) **P**_**5**_; (4) 1% (w/v) **P**_**1;**_ (5) 0.2% (w/v) **P**_**5**_; (6) 0.2% (w/v) **P**_**1**_; (7) 1% (w/v) **P**_**1,direct**_, (8) 0.2% (w/v) **P**_**1,direct**_. Source data

In principle, overlap domains could be designed with perfectly orthogonal sequences. However, this would require costly synthesis of many different oligonucleotides. Instead, we chose a combinatorial approach where overlap domains are diversified by introducing ambiguous bases (N) at specific positions (Fig. 1d). N indicates a position at which an equal mixture of A, T, C and G nucleotides is used during synthesis. The complexity (that is, the number of distinct splint pairs) of this combinatorial crosslinker library (CCL) equals 4^*n*^, where *n* is the number of ambiguous bases in the sequence. As the DNA duplex is strongly destabilized even by a single base mismatch^48^, equilibrium binding favours perfectly complementary binding partners. We carried out thermodynamic predictions for all splint combinations in libraries ranging from 1 (*n* = 0) to 256 (*n* = 4) splint pairs, confirming that highly selective splint pairing (>95%) is expected in all crosslinker libraries (Fig. 2b, Supplementary Fig. 5 and Supplementary Table 1).

## CCLs control network formation and matrix stiffness

Statistical simulations illustrate how CCL complexity and the number of anchor strands per polymer can affect crosslinking efficiency (Fig. 2a). To suppress 80% of intramolecular crosslinks, **P**_**1**_, **P**_**5**_ and **P**_**10**_ were predicted to require 4, 40 and 60 splint pairs, respectively. We first supplemented **P**_**5**_ with CCL libraries containing 1, 4, 16, 64 or 256 splint pairs (CCL-1 to CCL-256). Elastic moduli (*G*′) greatly exceeded the loss moduli (*G*″) over a wide range of frequencies and strains, confirming that dual-splint CCLs combined with UHMW polymers generate stable gels (Supplementary Fig. 6). Increasing CCL complexity gradually improved crosslinking efficiency from 28% (CCL-1) to 76% (CCL-64), with no further increase beyond the CCL-64 library size (Fig. 2c and Supplementary Note 2.3), which is in good agreement with the prediction (Fig. 2a and Supplementary Note 2.4).

The high crosslinking efficiency combined with UHMW polymer backbones enabled the formation of stable gels with remarkably low DNA content when compared with previously reported synthetic DNA gels with well-characterized rheological properties (Fig. 2d). CCL-64-crosslinked **P**_**1**_ and **P**_**5**_ showed critical gelation concentrations of ~0.2% (w/v), corresponding to a DNA content of 0.05 g l^−1^ and 0.26 g l^−1^, respectively. This concentration is close to the polymers’ apparent density (Supplementary Table 2), indicating that gelation occurs efficiently as soon as polymer blobs start overlapping in solution. Even lower DNA contents (down to 0.017 g l^−1^) were achieved when the CCL-64 overlap domain was directly grafted onto the polymer backbone (Fig. 2d, samples (7) and (8); Supplementary Fig. 10). The directly grafted approach offers a more efficient use of DNA, however, at the expense of ‘hard-coding’ the crosslinker properties into the polymer. We used crosslinker splints in all subsequent experiments, as they enable rapid adjustment of material properties without time-consuming polymer synthesis.

**Fig. 3:**
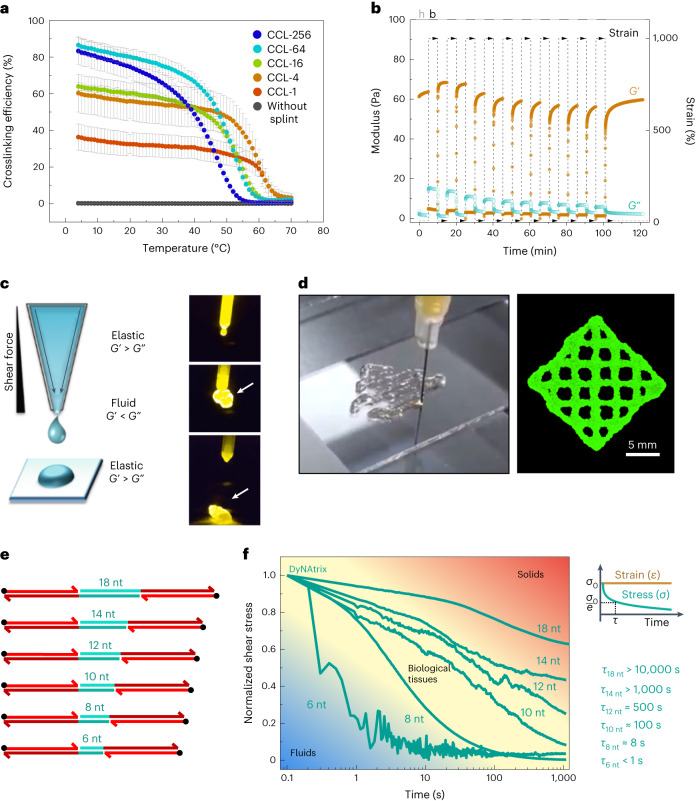
Thermoreversibility, self-healing, bioprinting and tunable stress-relaxation of DyNAtrix. **a**, Crosslinking efficiency of DyNAtrix (1% (w/v) **P**_**5**_ with different CCLs) as a function of temperature. Crosslinking efficiencies were calculated from the affine network model (Supplementary Note 2.3 and Supplementary Fig. 8). Data are shown as mean ± s.d. (*n* = 2). **b**, Self-healing of DyNAtrix (1% (w/v) **P**_**5**_ crosslinked with CCL-4) was confirmed by repetitive strain recovery tests alternating between 1,000% strain (breaking period, b) and 10% strain (healing period, h). When 1,000% strain is applied, DyNAtrix liquefies, as revealed by a drastic drop of its stiffness (*G*′ ≪ *G*″) and increase of the phase angle from 3° ± 1° to 74° ± 1°. DyNAtrix showed over 95% of recovery after 10 consecutive breakage–recovery cycles. **c**, Scheme of extrusion printing (left) and photographs of DyNAtrix being extruded from a narrow tip (right; the arrow marks the extruded gel; compare Supplementary Video 1). The gels were prepared with 1% (w/v) **P**_**5**_ crosslinked with CCL-64. **d**, A grid printed on a BioScaffolder with a total height of ~500 μm. The material was stained with SYBR Gold for imaging purposes. **e**, Scheme of SRCs with variable overlap domains (green). The corresponding DNA sequences are shown in Supplementary Table 1. **f**, Normalized stress-relaxation curves of DyNAtrix crosslinked via different SRCs (green traces) covering the typical range of biological tissues^21^ (yellow background). The shear stress (*σ*) was recorded over time at a fixed strain of 15%. Stress relaxation times (*τ*) were determined by the simple Maxwell model, *σ*(*τ*) = *σ*_0_/*e*, where *σ*_0_ is the shear stress measured at the earliest time point and *e* is Euler’s number. The measurements were carried out at 37 °C. Source data

As expected, DyNAtrix undergoes a thermally reversible sol–gel transition (Fig. 3a and Supplementary Fig. 8a). Its melting point decreases with increasing CCL complexity from 65 °C (CCL-1) to 53 °C (CCL-256), as the concentration of each distinct splint pair decreases with increasing library complexity. The observed shift in matrix melting is in excellent agreement with nearest-neighbour thermodynamic predictions for the overlap sequence (Supplementary Fig. 8b).

The well-defined reversibility of DyNAtrix*’*s bonds also facilitates bioprinting. Extrusion bioprinters require self-healing hydrogels that can be liquefied under mechanical stress, pass through the printer nozzle as a fluid while protecting cells from damaging forces, and quickly solidify after extrusion. To test printability, we performed self-healing experiments by oscillatory rheology: when subjected to large deformations, the supramolecular network breaks apart, but recovers rapidly once the stress is reduced (Fig. 3b and Supplementary Fig. 9). Over the course of 10 consecutive breakage–healing cycles, DyNAtrix restored 95% of its stiffness, indicating that the reversible crosslinks serve as pre-defined mechanical breakage points. Owing to its self-healing, DyNAtrix is easily extruded and printed through a thin nozzle (Fig. 3c,d and Supplementary Video 1). Embedded cells showed high viability and proliferation after printing, demonstrating that DyNAtrix is suitable for top-down fabrication of living tissue scaffolds (Supplementary Fig. 14).

## DNA sequences encode stress relaxation and heat activation

The force required to break a short (≤20 nt) DNA duplex into two separate strands depends on the sequence composition and grows linearly with the number of base pairs^49^. Exploiting this phenomenon, we developed stress-relaxation crosslinkers (SRCs) by systematically changing only a few bases in the overlap domain (Fig. 3e and Supplementary Table 1). As overlap domains are the weakest connections within the supramolecular network, their nanomechanical stability was expected to define the material’s macroscopic stress-relaxation characteristics. Indeed, SRCs with overlap domains ranging between 6 and 18 nucleotides allowed the adjustment of stress-relaxation times (*τ*) from less than one second to several hours (Fig. 3f). SRCs with 8 to 18 nucleotide overlap domains exhibit similar plateau stiffnesses, confirming that *G*′ and *τ* are independently tunable in DyNAtrix (Supplementary Fig. 7). Due to its subsecond relaxation time, gels crosslinked by 6-nucleotide SRCs are only solid at high deformation frequencies. Overall, SRCs allow precise mimicking of the typical stress-relaxation times that are found across diverse animal tissues^24^.

**Fig. 4:**
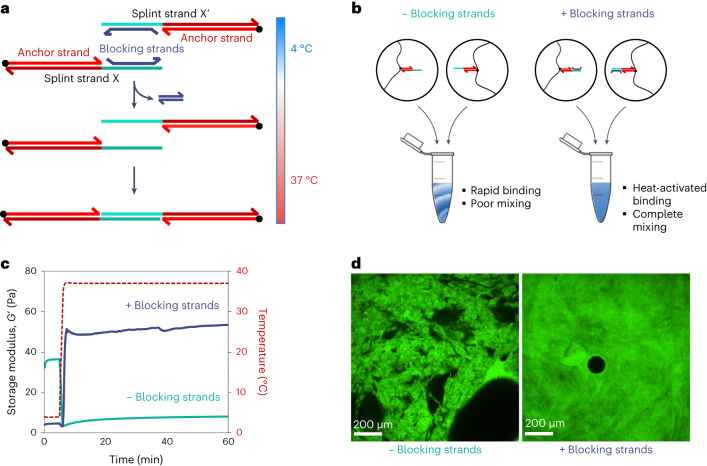
HACs enable controlled gelation of DyNAtrix under cell-compatible conditions. **a**, Scheme of HACs and their binding mechanism. **b**, Scheme of liquid precursor mixing with versus without blocking strands. **c**, Storage modulus *G*′ of the gels (1% (w/v) **P**_**5**_ + CCL-64), directly after mixing, with (blue) and without (green) blocking strands. **d**, Confocal microscopy images (cross-section) of DyNAtrix (1% (w/v) **P**_**5**_ + CCL-64) formed with and without blocking strands. One polymer component was stained with a fluorescein-labelled DNA strand (green fluorescence), while the other was unlabelled. Left: poor mixing is typically observed in the absence of blocking strands. Right: homogeneous molecular networks are observed with HACs (+ blocking strands). The black circle is an air bubble. Source data

The next goal was to render DyNAtrix compatible with standard cell encapsulation processes. In classical 3D culture, cells are dispersed in a precursor solution, and subsequently gelation is initiated. For instance, Matrigel solution and cells are mixed at 4 °C, followed by heating to 37 °C, which triggers gelation at the ideal temperature for mammalian cell culture. In contrast, liquefying DNA-crosslinked gels typically requires heating beyond the crosslinker melting temperature, which would be damaging to cells. We initially attempted to overcome this problem by encapsulating cells via rapid mixing of two pre-annealed precursor solutions (Fig. 4b, left). However, the fast binding kinetics led to rapid crosslink formation before mixing could be completed, creating an inhomogeneous matrix with inadequate stiffness (Fig. 4c and Fig. 4d, left).

As a solution, we designed heat-activated crosslinkers (HACs) by upgrading the CCL design with blocking strands (Fig. 4a and Supplementary Note 2.5). Blocking strands serve as protection groups that prevent premature overlap domain binding at low temperatures. A mixture of two polymer precursor solutions is thus trapped in a metastable liquid state at 4 °C, such that the mixing of cells and medium can proceed to completion. The blocking strands were designed to spontaneously dissociate from the overlap domains at 37 °C, thereby activating the crosslinker. In agreement with this design, heating to 37 °C led to rapid formation of a homogeneous gel (Fig. 4d) with superior stiffness (Fig. 4c). HACs were subsequently used in all cell-culture experiments.

## High stability, tunable degradation and biocompatibility

**Fig. 5:**
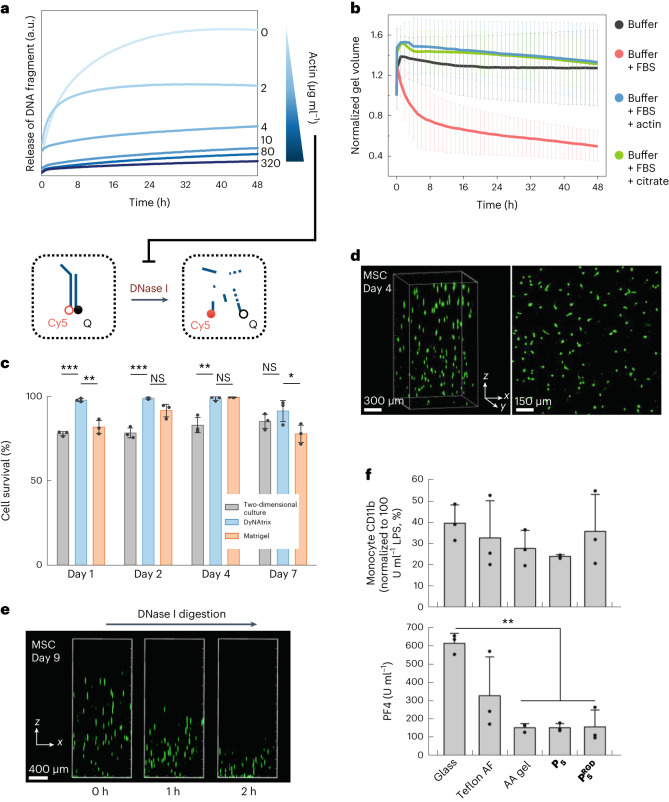
DyNAtrix exhibits tunable stability against degradation in cell culture, high biocompatibility and DNase I-mediated release of live cells. **a**, Digestion experiment. A digestion probe containing a fluorophore (Cy5) and a fluorescence quencher (Q) was incubated in medium containing DNase I and different concentrations of actin. Duplex digestion leads to dissociation of the strands and increased Cy5 emission. **b**, Normalized volume of DyNAtrix in FBS-supplemented medium over time. Samples were incubated in buffer (negative control; black) or serum-containing medium without protection (red). The addition of actin (50 µg ml^−1^; blue) or citrate (10 mM; green) effectively protected the gels from digestion. Data are shown as mean ± s.d. (*n* = 4). **c**, Viability of MSCs cultured in actin-protected DyNAtrix compared with Matrigel and a two-dimensional culture plate. Data are shown as mean ± s.d. (*n* = 3). Statistical analysis was performed using one-way ANOVA followed by a Tukey post hoc test. NS, not significant; *P < 0.05; **P < 0.01; ***P < 0.001. **d**, Live/dead staining of MSCs in DyNAtrix (1% (w/v) ${{\bf{P}}}_{{\bf{5}}}^{{\bf{R}}{\bf{G}}{\bf{D}}}$ + CCL-64) after 7 days in culture. Live cells were stained with calcein AM (green) and dead cells were stained with propidium iodide (pink). **e**, Release of live MSCs from DyNAtrix under mild conditions via the addition of DNase I on day 9. **f**, Quantification of DyNAtrix-triggered immune response and haemocompatibility. Top: activation of monocytes in human whole blood was measured by flow cytometric quantification of CD11b expression. Bottom: platelet activation was assessed by quantification of the haemostasis marker platelet factor 4 (PF4). CCL-64-crosslinked 1% (w/v) **P**_**5**_ and ${{\bf{P}}}_{{\bf{5}}}^{{\bf{R}}{\bf{G}}{\bf{D}}}$ were compared with 3 reference substrates: reactively cleaned glass, polytetrafluoroethylene (Teflon AF) and covalently crosslinked polyacrylamide (AA) gel. Data are shown as mean ± s.d. (*n* = 3). Statistical analysis was performed using one-way ANOVA followed by a Holm–Sidak post hoc test (**P < 0.01). Source data

DNA-based gels are susceptible to unintended digestion by DNase I, a nuclease commonly found in serum-supplemented media. We aimed to protect DyNAtrix yet preserve the option for controlled enzymatic modification whenever needed. We first tested the stability of DyNAtrix in the presence of fetal bovine serum (FBS)-supplemented culture medium, revealing that substantial degradation indeed took place within 48 h (Supplementary Video 2). We then tested the effect of actin, which is known to inhibit DNase I (ref. ^50^). Using a fluorescently labelled DNA mock target, we showed that actin can be used to tune the rate of digestion by DNase I (Fig. 5a). A concentration of 50 µg ml^−1^ was sufficient to suppress gel degradation for 48 h (Fig. 5b and Supplementary Video 3) and 80 µg ml^−1^ provided near-complete DNase I inhibition (Supplementary Fig. 25), preserving DyNAtrix in cell-culture experiments for at least 2 weeks. A similarly effective protection was achieved by the addition of citrate. However, as chelation of calcium ions by citrate was suspected to affect cell development, we chose actin as our default protection strategy.

To examine cytocompatibility, we cultured human mesenchymal stromal cells (MSCs) in actin-protected DyNAtrix. MSCs showed excellent viability over the course of 7 days (91–99%), significantly higher compared with a two-dimensional culture dish, and even outperforming Matrigel in most experiments (Fig. 5c and Supplementary Fig. 12). The subsequent addition of recombinant DNase I overwhelmed the actin protection, allowing gradual matrix deconstruction and on-demand release of live cells (Fig. 5e and Supplementary Video 4).

In assessing DyNAtrix’s biomaterial properties, we incubated HAC-64-crosslinked **P**_**5**_ and ${{\bf{P}}}_{{\bf{5}}}^{{\bf{R}}{\bf{G}}{\bf{D}}}$ with human whole blood and compared inflammation and haemostasis markers with the three reference substrates glass, Teflon AF (a benchmark material for medical devices) and covalently crosslinked polyacrylamide. DyNAtrix showed low activation of monocytes (Fig. 5f), granulocytes (Supplementary Fig. 11b,c) and the complement system (Supplementary Fig. 11a), exhibiting no significant increase in innate immune response compared with the reference substrates. Both gels demonstrated reduced blood coagulation and platelet activation compared with glass and Teflon AF, but similar to polyacrylamide (Fig. 5f and Supplementary Fig. 11d). No statistically significant differences were observed between gels constructed from **P**_**5**_ and ${{\bf{P}}}_{{\bf{5}}}^{{\bf{R}}{\bf{G}}{\bf{D}}}$. Overall, the results corroborate the potential suitability of DyNAtrix for in vivo studies and medical applications.

## DyNAtrix supports development of diverse cells and organoids

**Fig. 6:**
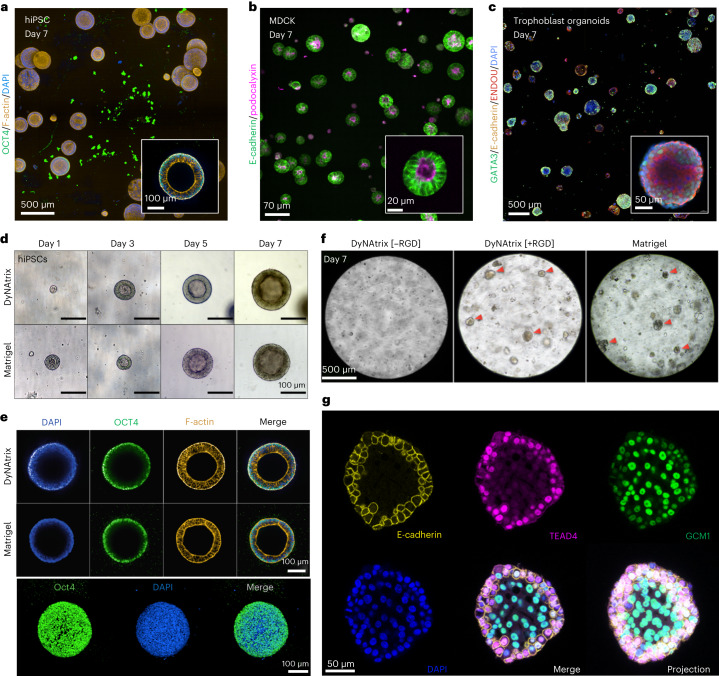
DyNAtrix is compatible with a variety of 3D cell and organoid cultures. **a**, Confocal image of hiPSC cysts embedded in DyNAtrix [+RGD] that was crosslinked in serum-free mTeS1 medium. Cells were stained for OCT4 (pluripotency), phalloidin (F-actin) and DAPI (nuclei). **b**, Confocal image of MDCK cysts grown in DyNAtrix [+RGD] using heat-activated 18 nt SRCs as the crosslinker library. The cells were modified to express mNeonGreen-labelled E-cadherin and mScarlet-labelled podocalyxin. **c**, Confocal image of trophoblast organoids cultured in DyNAtrix [+RGD]. Cells were immunofluorescently stained for GATA3, E-cadherin, ENDOU and DAPI (nuclei). **d**, Representative microscopy images of hiPSC cysts grown in DyNAtrix [+RGD] compared with Matrigel. A comparison with DyNAtrix [−RGD] is shown in Supplementary Fig. 15. **e**, Representative confocal microscopy images of hiPSC cysts grown in DyNAtrix [+RGD] compared with Matrigel (day 7). Top: cross-sections. Bottom: projection image of a cyst in DyNAtrix [+RGD]. **f**, Representative bright-field images of trophoblast organoids grown in DyNAtrix [+RGD]/[–RGD] versus Matrigel. The red arrows mark organoids. **g**, Confocal microscopy images of a trophoblast organoid in DyNAtrix [+RGD] stained for TEAD4, E-cadherin, GCM1 and DAPI (nuclei) (day 7). A comparison with organoids in Matrigel is shown in Supplementary Fig. 21.

To validate the applicability for advanced cell culture and organoid research, three additional cell types were embedded in DyNAtrix: (1) human induced pluripotent stem cells (hiPSCs), which offer patient-specific platforms for in vitro disease modelling at the cellular level (Fig. 6a,d,e), (2) Madin–Darby canine kidney (MDCK) cells, a model system for developing epithelial tissues (Fig. 6b), and (3) human trophoblast stem cells (hTSCs) that develop into trophoblast organoids, allowing in vitro studies of placental development and pregnancy-related disorders (Fig. 6c,f,g). We assessed viability, proliferation and self-organization of the cells and tested the effect of adhesion sites ([+RGD] versus [−RGD]) on their development and morphogenesis.

hiPSCs were cultured in the presence of a chemically defined serum-free medium. Notably, DyNAtrix stayed intact for the full culture duration (7 days) even without actin supplementation. Similar results were obtained in serum-free MDCK and trophoblast cultures (Fig. 6b,c), suggesting that the main source of nucleases is supplemented serum, rather than secretion by the embedded cells. hiPSC viability in DyNAtrix and Matrigel were statistically indistinguishable (Supplementary Fig. 13a). During the culture for 1 week, hiPSCs sustained continuous proliferation, formed hollow cysts that remained round-shaped, and continued expanding (Fig. 6d). The size, morphology and number of cysts appeared identical to those formed in a Matrigel reference culture. Confocal microscopy showed that the cysts strongly expressed pluripotency marker OCT4, further demonstrating that the material is effectively supportive (Fig. 6e). The presence of mechanical cell-matrix connection points proved critical, as the number of cysts and their lumen sizes were greatly reduced in DyNAtrix[−RGD] (Supplementary Fig. 15).

We were initially sceptical whether staining for nuclear DNA in cells was feasible, as the DNA crosslinkers were suspected to create a high extracellular background signal. However, due to the low crosslinker concentration, cell nuclei were readily stained in all experiments (Fig. 6a,e,g). Background fluorescence was observed in a few instances (Supplementary Fig. 16), but did not affect nuclear staining or imaging.

The culture of MDCK cysts largely mirrored the results from hiPSC culture. In serum-free medium, DyNAtrix remained stable without actin protection for at least 2 weeks. MDCK cells were genetically modified to express fluorescent podocalyxin and E-cadherin as markers for cell polarity and adherens junctions. Confocal microscopy showed that MDCK cells proliferate, form hollow cysts and exhibit very high viability (Supplementary Fig. 13b). Cyst morphologies strongly depended on the presentation of extracellular adhesion signals: in DyNAtrix[−RGD], only 2% (±1%) of cysts showed an apical-in polarity, while the majority exhibited an inverted or undetermined polarity. In contrast, 20% (±1%) of cysts grown in DyNAtrix[+RGD] showed apical-in morphologies (Supplementary Fig. 17). Intriguingly, cyst polarity could be further modulated via the choice of the material’s stress relaxation. Replacing the default 14 nt SRC with the 18 nt SRC increased the apical-in polarity to 90% (±6%) (Fig. 6b). The dependence of MDCK cyst polarity on stress relaxation underlines the importance of mechanically programmable viscoelastic matrices to guide morphogenesis. A detailed study on the effects of stress relaxation on epithelial cell polarity in DyNAtrix is currently ongoing.

We finally explored the development of trophoblast organoids from hTSCs in DyNAtrix. We chose this particular organoid, as the adjustable viscoelasticity and biochemical cues could solve current challenges surrounding organoid polarity to more faithfully reproduce in vivo morphologies^51^. In DyNAtrix[−RGD], trophoblast proliferation was low, and no organoids were observed (Fig. 6f). In contrast, trophoblasts in DyNAtrix[+RGD] proliferated and developed numerous organoids within one week. The number, size, shape and viability of the organoids closely matched those grown in Matrigel (Fig. 6f and Supplementary Figs. 13c and 18). To demonstrate sustained proliferation, we adapted a recently reported protocol for long-term trophoblast organoid culture^51^. Digestion of DyNAtrix crosslinkers by addition of recombinant DNase I allowed the release and successive passaging of organoids, resulting in a total culture duration of up to 21 days (Supplementary Figs. 23 and 24). Confocal imaging revealed strong expression of GATA3 (GATA binding protein 3), a trophoblast-specific transcription factor typically found in all cells of trophoblast organoids (Fig. 6c). Moreover, we confirmed the expression of GCM1 (glial cells missing transcription factor 1), TEAD4, (TEA domain transcription factor 4) syndecan, ENDOU (endonuclease, poly(U) specific) and E-cadherin, which are important markers for cell differentiation and trophoblast organoid development^52,53^ (Fig. 6g and Supplementary Figs. 19–22). Overall, the experiments showed no significant differences between organoids in DyNAtrix and Matrigel, demonstrating that human trophoblast organoids can be grown in a fully synthetic 3D matrix environment.

## Conclusions

Our results illustrate the power of DNA nanotechnology in soft materials engineering towards programmable, adaptive, self-healing and printable 3D cell-culture matrices. The key feature of DyNAtrix is its potential for rational material design, permitting researchers to translate predictable molecular properties into macroscopic material characteristics: the complexity of CCLs determines network formation (Figs. 1c and 2a), presenting a fundamentally new way of optimizing matrix elasticity without changing polymer or crosslinker concentrations (Fig. 2c). SRCs encode (nano)mechanical stability (Fig. 3), allowing systematic adjustment of stress relaxation by altering merely a few bases on the crosslinker sequence. HACs show customizable binding kinetics (Fig. 4), which is critical for homogeneous cell encapsulation and seamless integration with existing cell-culture workflows. This high level of control helps mimic many of the properties of complex biological matter, but in a fully synthetic and compositionally defined material, improving reproducibility and reducing regulatory hurdles in regenerative medicine.

Previous attempts at DNA-based soft materials faced challenges such as high-DNA-content requirements and premature gel degradation. Our approach combines a UHMW polymer backbone with crosslinker libraries, allowing much lower DNA concentrations. This results in soft gels with suitable elastic moduli for many mechanosensitive stem cells and organoid systems^54^. Due to its low DNA content, DyNAtrix is non-immunogenic, cost-effective (Supplementary Table 4) and potentially applicable for in vivo use cases, for instance, as injectable cell-laden gels, drug release systems or coatings for medical devices.

Actin protection—or the use of serum-free media—allows cell culture for prolonged periods of time. Notably, trophoblast organoids were successfully cultured in DyNAtrix for up to 3 weeks. Achieving such long culture time is critical for future studies on many other human organoid systems. Despite its high stability, DyNAtrix is readily deconstructed on demand. In particular, its nuclease/actin-regulated degradation represents an externally controlled alternative to the commonly relied-on matrix degradation by cell-secreted metalloproteinases.

In summary, DyNAtrix offers a mix-and-match approach to matrix engineering. DNA modules and polymer backbones with different properties can be deployed in various combinations. For example, a HAC-crosslinked DyNAtrix can be further upgraded with fluorescent DNA stress sensors^55,56^ to shed light onto the mechanical interactions of cells with their environment. DNA aptamers can be integrated to scavenge (or gradually release) specific biomolecules. Such modules can be activated on demand (for example, by temperature), modified (for example, by hybridization) and subsequently released off the polymer scaffold (for example, by restriction enzymes). The effects of stress-relaxation times on the development of various cell and organoid models are currently studied in our lab. In the future, DyNAtrix could be autonomously controlled by molecular logic gates and synthetic regulatory circuits based on DNA strand displacement cascades^10,57^. We therefore envision this class of programmable materials to create unprecedented options for 3D cell and organoid culture and a range of other biomedical applications.

## Methods

### Materials

Solvents and reagents were purchased from commercial sources and used as received, unless otherwise specified. Methanol (ACS reagent grade) was obtained from Fisher Scientific. Molecular biology grade acrylamide (catalogue number A9099), sodium acrylate (catalogue number 408220), 19:1 acrylamide/bis-acrylamide (catalogue number A2917) and ammonium persulfate (APS; catalogue number A3678) were purchased from Sigma-Aldrich. Ultrapure *N*,*N*,*N*′,*N*′-tetramethylethylenediamine (TEMED; catalogue number 15524010) and SYBR Gold Nucleic Acid Gel Stain (catalogue number S11494) were obtained from Thermo Scientific. Desalted oligonucleotides were purchased from Integrated DNA Technologies (IDT). Nitrogen gas (>99.999%) was used under inert conditions and supplied by an in-house gas generator. To ensure an inert condition, it was purified through a Model 1000 oxygen trap from Sigma-Aldrich (catalogue number Z290246). Reagents with unreacted acrylamide groups were stored at 4 °C or −20 °C, protected from unnecessary exposure to light. Actin protein (>95% pure, rabbit skeletal muscle, catalogue number AKL95) was obtained from Cytoskeleton and DNase I (catalogue number M0303) was obtained from New England Biolabs. Matrigel was obtained from Corning (catalogue numbers 354277 and 356231). DNA ladders were purchased from Thermo Fischer (catalogue number SM1211).

### Cell sources

Human bone marrow-derived MSCs were isolated from healthy female and male donors (aged 26–37) with informed consent by the Medical Faculty at the University Hospital Dresden (Ethikkommission an der Technischen Universität Dresden, ethic board number EK263122004). MDCK II cells (ECACC 00062107) were provided by A.H.’s group. Human pluripotent stem cells were generated at the CRTD Stem Cell Engineering Facility, Technische Universität Dresden, by Shahryar Khattak and registered under the hPSCreg name CRTDi003-B (https://hpscreg.eu/cell-line/CRTDi003-B). The CT27 patient-derived trophoblast stem cell (TSC) line used in this study was obtained from the RIKEN stem cell bank (https://cellbank.brc.riken.jp/cell_bank/CellInfo/?cellNo=RCB4936). The CT27 TSCs were derived from placental cytotrophoblast cells as described in ref. ^62^. Human placentas were obtained from healthy women with signed informed consent of the donors, and the approval of the Ethics Committee of Tohoku University School of Medicine (Research license 2014-1-879).

### Polymer synthesis

A detailed protocol is available in ref. ^45^. In brief, acrylamide (50 mg ml^−1^), sodium acrylate (0.5 mg ml^−1^) and acrylamide-labelled anchor strand DNA (Supplementary Table 1) were co-polymerized in TBE buffer (100 mM Tris, 100 mM boric acid, 2 mM EDTA, pH 8.2). For **P**_**1**_, **P**_**5**_ and **P**_**10**_, the DNA concentrations in solution were 100 µM, 500 µM and 1,000 µM, respectively. For the corresponding RGD-functionalized derivatives ${{\bf{P}}}_{{\bf{5}}}^{{\bf{RGD}}}$ and ${{\bf{P}}}_{{\bf{10}}}^{{\bf{R}}{\bf{G}}{\bf{D}}}$, 10 mM of acrylated RGD peptide (sequence, G(acryl-K)GGGRGDSP) was co-polymerized with the above solution. Polymerization reactions for **P**_**1**_, **P**_**5**_ and ${{\bf{P}}}_{{\bf{5}}}^{{\bf{RGD}}}$ were initiated with 0.005 wt% APS in presence of 0.005 wt% TEMED. Synthesis of **P**_**10**_ and ${{\bf{P}}}_{{\bf{10}}}^{{\bf{R}}{\bf{G}}{\bf{D}}}$ was initiated by 0.025 wt% TEMED and 0.025 wt% APS. To achieve high molecular weight and a narrow size distribution, it was necessary to carry out the reaction in high-purity nitrogen gas, which was passed through an oxygen trap on-site. The reaction was allowed to proceed overnight, resulting in a highly viscous polymer solution indicating the formation of long polymer chains. NMR spectroscopy was used to verify high conversion of the monomer (Supplementary Fig. 3). The solution was diluted in 9 volumes of TE buffer (10 mM Tris, 1 mM EDTA, pH 8.0) and subsequently purified via methanol precipitation. The pellet was resuspended in milliQ water and stored in aliquots at −20 °C. A small aliquot of the polymer solution was lyophilized and weighted to determine the synthesis yield. Yields: 89% (**P**_**1**_), 98% (**P**_**5**_), 93% (**P**_**10**_), 92% (${{\bf{P}}}_{{\bf{5}}}^{{\bf{RGD}}}$) and 88% (${{\bf{P}}}_{{\bf{10}}}^{{\bf{R}}{\bf{G}}{\bf{D}}}$).

### Nearest-neighbour thermodynamic predictions

Explicit sequences of crosslinking strands and their complements were generated using a custom Python script. The program sequentially replaces the ambiguous bases N with nucleobases (A, C, G, T) via multilayer nested loops. This created a library of forward and reverse splint variants. The minimum free energies (MFEs) of all explicit overlap domains were calculated against all explicit overlap domains in the library using NUPACK (version 4.0.0.27), using nearest-neighbour thermodynamic parameters according to ref. ^63^. The model parameters were set to *T* = 20 °C, 150 mM NaCl, 75 µM total DNA concentration (37.5 µM for each splint strand), and a complex size of 2. For each possible pair, the Boltzmann distribution factor *B*_*x*,*y*_ was sequentially calculated for each variant of forward (*x*) against all possible reverse splint (*y*) strand at the expected theoretical concentration using the equation

$${B}_{x,y}={\rm{e}}^{-{{\rm{MFE}}(x,y)}/{kT}}$$

where *k* is the Boltzmann constant. The normalized distribution was calculated to study the selective affinity of any forward splint variant in a pool of reverse splint sequences. This was calculated for each pair as the ratio of distribution factor of one pair (*x*, *y*) by the sum of all pairs as

$${B}_{x,y}^{{\mathrm{normalized}}}=\frac{{B}_{x,y}}{{\sum }_{y}{B}_{x,y}}$$

We note that a precise solution can only be obtained by numerically solving a system of ordinary differential equations^63^. For simplification, we assumed that each splint strand is available at its initial concentration. In reality, non-complementary splint strand concentrations are expected to be significantly reduced, as they would be predominantly scavenged by their complementary binding partners. The obtained values for specific CCL pairs in the Boltzmann distribution that are based on our simplified calculation are therefore considered a lower limit for relative abundances.

### Thermal annealing of DNA-crosslinked hydrogels

Unless specified, the samples were prepared at the final concentration of 1% (w/v) polymer solution, added with one equivalent of DNA crosslinkers to the anchor strand (for example, 75 μM crosslinkers for 1% (w/v) **P**_**5**_ solution), in the buffer condition of 150 mM NaCl and 1x TE buffer. The samples were annealed on the thermal cycler using the following steps: (1) heating at 95 °C for 3 min, (2) instant cooling from 95 °C to 80 °C, (3) holding at 80 °C for 2 min, (4) first cooling ramp from 80 °C to 65 °C at −0.3 °C min^−1^, (5) second cooling ramp from 65 °C to 37 °C at −0.5 °C min^−1^, (6) holding at 37 °C for 24 h.

The first cooling ramp facilitates binding of the adaptor domains of the crosslinkers to the anchor strands, while the second ramp allows proper binding of the overlap domains to their complementary partners.

### Oscillatory rheological measurements

Viscoelasticity measurements were conducted using Anton Paar MCR301 rheometer with a 25-mm-diameter cone-plate geometry (cone angle 0.5°). Temperature control was ensured by installing a Peltier system PTD-200 (Anton Paar) within the measurement area. To maintain high humidity and prevent evaporation artefacts, a wet paper cylinder was placed around the plate’s circumference. Amplitude sweeps were performed from 0.1% to 1,000% strain at 1.6 Hz, while frequency sweeps were conducted from 0.1 Hz to 100 Hz at 10% strain. Temperature sweeps covered a range from 4 °C to 60 °C at 10% strain, followed by 60 °C to 70 °C at 20% strain, all at 1.6 Hz. The heating and cooling steps were performed reversibly for two cycles to exclude evaporation artefacts. Unless specified, samples were measured in triplicate for reproducibility. Stress-relaxation properties were measured at 15% strain, where the strain was kept constant while recording shear stress over time. Self-healing tests involved alternating between 1,000% and 10% strain for 5 min each, repeated 4.5 times at a frequency of 1.6 Hz. For the heat-activated gelation tests, two precursors were prepared at 1% (w/v) polymer solution, added with: (1) 40 µM forward CCL-4 strands (strand ID 4a) and 80 µM corresponding blocking strand (strand ID 14a), and (2) 40 µM reverse CCL-4 library (strand ID 4b) and 80 µM corresponding blocking strand (strand ID 14b). The precursors were heated at 95 °C for 3 min, slowly annealed with a cooling ramp from 95 °C to 4 °C at −3 °C min^−1^, and kept at 4 °C overnight. Before measurement, the two precursors were mixed and applied onto the rheometer. The samples were held at 4 °C for 5 min, quickly heated to 37 °C and maintained at 37 °C for 60 min with shearing at 10% strain and a frequency of 1.6 Hz.

### Hydrogel printing

A microscope glass slide (76 × 26 mm, Thermo Scientific) was attached to the printing bed on BioScaffolder BS5.1 (GeSiM). Two-hundred microlitres of DyNAtrix (1% (w/v) **P**_**5**_ + CCL-64) stained with SYBR Gold was transferred to a disposable 1 ml syringe (Omnican 40). To remove air bubbles, the syringe was centrifuged invertedly at 300*g* for 2 mins. The hydrogels were printed through a 30-gauge needle (Omnican) at room temperature, with a printing speed of 2 mm s^−1^, an extrusion rate of 40 μm s^−1^ and a layer height of 0.12 mm. The resulting 3D structures, with a size of 20 × 20 × 2 mm (*L* × *W* × *H*), were imaged on Opera Phenix confocal microscope (PerkinElmer) at ×5 magnification.

### Mixing homogeneity tests

Two precursor solutions were prepared in 1x TE buffer at the final concentration of 1% (w/v) **P**_**5**_ added with: (1) 40 µM forward CCL-64 strands (strand ID 6a) and 80 µM corresponding blocking strand (strand ID 15a), and (2) 40 µM reverse CCL-64 library (strand ID 6b) and 80 µM corresponding blocking strand (strand ID 15b). Ten micromolar of a 6-FAM (fluorescein)-modified strand (strand ID 15) was added to the precursor (1). After 1 h of equilibration at 4 °C, the two precursors were mixed using a positive displacement pipette. The mixture was dispensed into a 384-well plate (Greiner Bio-One) at a volume of 10 µl per well. The plate was then incubated at 37 °C for 30 min to initiate gelation. Finally, images were acquired on Andor Dragonfly Confocal Microscope (Oxford Instrument) at ×4 magnification.

### Enzymatic digestion assay

The Förster-resonance-energy-transfer-paired oligos, modified with Cy5 fluorophore and Iowa Black Dark quencher (Q), were purchased from IDT. A mixture of 2 µM Cy5-strand (strand ID 13a) and 4 µM Q-strand (strand ID 13b) was prepared in 1x PBS buffer and subjected to annealing steps: heating at 95 °C for 1 min, instant cooling to 50 °C for 2 min and gradual cooling from 50 °C to 20 °C at a rate of −1.5 °C min^−1^. Samples were prepared with a final concentration of 100 nM Cy5-Q Förster-resonance-energy-transfer-probe and varying actin content from 2.5 µg ml^−1^ to 320 µg ml^−1^ (Cytoskeleton, catalogue number AKL95-B) in a buffer containing 150 mM NaCl, 1.8 mM CaCl_2_, 0.2 mM ATP (Jena Bioscience, catalogue number NU-1010) and 1x IDTE. Before measurement, 80 U ml^−1^ DNase I (NEB, catalogue number M0303S) was added to each sample. Each sample was loaded in triplicate at a volume of 20 µl per well into a 96-well quantitative PCR plate. Fluorescence signals were recorded every 10 min for 24 h, and subsequently every 30 min for the following 36 h at 37 °C using a Bio-Rad CFX96 Real-Time PCR System.

### Gel volume and digestion measurements

The lyophilized actin protein (Cytoskeleton, catalogue number AKL95-B) was reconstituted with 100 µl deionized water to a concentration of 10 mg ml^−1^. The reconstituted actin was then in a buffer containing 5 mM Tris-HCl pH 8.0, 0.2 mM CaCl_2_, 0.2 mM ATP, 5% (w/v) sucrose and 1% (w/v) dextran. Citric acid powder (192.124 MW) was prepared in 1x PBS to a final concentration of 200 mM, pH 7.0. The gel (1% (w/v) **P**_**5**_ crosslinked with CCL-64) were stained with 10X SYBR Gold before swelling. The gel was then dispensed into a 384-well plate (Greiner, number 788092) at a volume of 5 µl per well. The samples were immersed in 20 µl of (1) 1x TE buffer, (2) 10% FBS-containing DMEM, (3) 50 µg ml^−1^ actin + 10% FBS-containing DMEM, or (4) 10 mM citrate + 10% FBS-containing DMEM. Images were captured every hour for 48 h at 37 °C on Opera Phenix Plus confocal microscope (PerkinElmer) at ×5 magnification. The gel volume was quantified using IMARIS software (Oxford Instrument) through the surface wizard and statistical tool under a uniform threshold setting.

### Whole blood incubation

Whole blood incubation was performed as described previously^64,65^. Gels were prepared on glass carriers with a polyethylene-*alt*-maleic anhydride bonding layer^66^, and inserted in in-house developed incubation chambers made of polytetrafluoroethylene, where a 3.2 cm^2^ test surface was exposed to 2 ml blood^64^. Reactively cleaned glass^67^ and Teflon AF (DuPont) served as activating and inert reference surfaces, respectively.

Blood was obtained from two voluntary ABO-matched donors who had not used any medicine in the past ten days. The blood was immediately anticoagulated with 1.0 U ml^−1^ heparin (Ratiopharm), pooled and filled into the incubation chambers with the samples, avoiding an air interface. The chambers were incubated for 2 h at 37 °C at constant overhead rotation to prevent sedimentation. The blood was subsequently analysed for the blood cell count (Beckmann Coulter AcTdiff). Granulocyte and monocyte activation were determined by flow cytometry (LSR Fortessa, Becton Dickinson), where granulocytes and monocytes were identified for their scatter characteristics and CD15 (clone SSEA-1, PE-conjugated, BioLegend) and CD14 (clone M5E2, APC conjugated, Becton Dickinson) positivity, respectively. The intensity of CD11b expression (clone ICRF44, PacificBlue-conjugated, BioLegend) was normalized to blood incubated with 100 EU ml^−1^ endotoxin. The rate of granulocytes with conformationally activated CD11b was determined using anti-CD11b clone CBRM1/5 (PE/Cy7-conjugated, BioLegend).

Soluble markers of coagulation activation (prothrombin fragment F1+2), blood platelet activation (platelet factor 4 (PF4)) and complement activation (complement fragment C5a) were determined from plasma using commercial enzyme-linked immunosorbent assays (ELISAs ; Enzygnost F1+2, Siemens Healthineers; Zymutest PF4, Hyphen BioMed; C5a ELISA, DRG Instruments) after stabilization of the blood with the recommended additives of the test kits and centrifugation.

The analysis was performed with a triplicate set of samples in parallel (*n* = 3). The study was covered by the ethic vote EK-BR-24/18-1 of the Sächsische Landesärztekammer.

### 3D cell culture

A detailed protocol is available in Supplementary Methods. In brief, gel components were dissolved in water and stored at −20 °C for cell culture. For MSC and hiPSC cultures, two precursors were prepared at a final concentration of 1% (w/v) ${{\bf{P}}}_{{\bf{5}}}^{{\bf{RGD}}}$ with: (1) 37.5 µM forward splint (strand ID 6a) and 75 µM blocking strand (strand ID 15a), and (2) 37.5 µM reverse splint strands (strand ID 6b) and 75 µM blocking strand (strand ID 15b). As for MDCKII and hTSC cultures, two precursors were prepared at a final concentration of 1% (w/v) ${{\bf{P}}}_{{\bf{10}}}^{{\bf{RGD}}}$ with: (1) 75 µM forward splint (strand ID 6a) and 150 µM blocking strand (strand ID 15a), and (2) 75 µM reverse splint strands (strand ID 6b) and 150 µM blocking strand (strand ID 15b). Concentrated DMEM was added to reach a final 1x concentration. The precursors were equilibrated at 4 °C overnight for the blocking strands to bind quantitatively to the splint strands.

The cell suspension was mixed with the first precursor (1) and then with the second precursor (2). Cell-laden hydrogels (5 µl per well) were placed in 384-well plates or micro-well inserts (ibidi, catalogue number 80409) attached in 6-well plates. The samples were incubated at 37 °C for 30 min to trigger heat-activated gelation. After gelation, individual culture medium was added to each well. When using serum-containing medium, 100 µg ml^−1^ of actin (Cytoskeleton, catalogue number AKL95) was included to suppress nuclease activity. The embedded cells were cultured at 37 °C, 5% CO_2_.

For Matrigel groups, cells were embedded in 50% Matrigel. The cell seeding density, gelation time and medium were identical to the DyNAtrix samples.

### Fluorescence live/dead assay and cell release

MSCs were stained with 3 µM calcein AM (PromoKine, catalogue number PK-CA707-80011) and 0.75 µM Draq7 (Invitrogen, catalogue number D15106) in medium for 30 min at 37 °C, 5% CO_2_. Confocal images were taken on Opera Phenix Plus confocal microscope (PerkinElmer) at ×10 magnification. Cell counting was performed with IMARIS software (Oxford Instrument) using the spots wizard. Cell viability was determined by dividing the number of live cells by the sum of live and dead cells.

For cell release, 2 U per well DNase I was added after the MSCs were stained with 3 µM calcein AM for 30 min. Confocal images were acquired every 20 min at ×5 magnification for 2 h at 37 °C with 5% CO_2_.

### Immunofluorescent staining

hiPSCs were fixed with 2% paraformaldehyde for 40 min at room temperature, followed by permeabilization and blocking with 0.1% Triton X-100 in 2% BSA/PBS for 1 h °C. The cells were then incubated overnight at 4 °C with the primary antibodies (1:300 anti-Oct3/4 (BD Biosciences, catalogue number 611202)) in 2% BSA/PBS. After washing the samples three times with PBS, secondary antibodies (1:200 Alexa Fluor 488, 1:200 Phalloidin ATTO 550 and 1:1,000 Hoechst 33342) in 2% BSA/PBS were added and incubated overnight at 4 °C. Confocal images were acquired using Opera Phenix Plus confocal microscope (PerkinElmer).

As for hTSCs and trophoblast organoids, the samples were fixed with 2% paraformaldehyde for 40 min at 4 °C, permeabilized with 0.5% Tween-20/PBS for 30 min and blocked with 3% BSA (Sigma) + 0.1% Tween-20 for 1 h. The cells were incubated with the primary antibodies in the blocking solution for 2 days at 4 °C. The primary antibodies used were: anti-E-cadherin (Invitrogen, catalogue number 13-1900, 1:200), anti-Syndecan (Sigma, catalogue number HPA006185, 1:200), anti-GATA3 (R&D, catalogue number AF2605, 1:200), anti-ENDOU (Sigma, catalogue number HPA012388, 1:200), anti-GCM1 (Atlas, catalogue number HPA011343, 1:200) and anti-TEAD4 (abcam, catalogue number ab58310, 1:200). After washing with PBS 3 times, the cells were then incubated with the secondary antibodies in the blocking solution for 2 days at 4 °C. The secondary antibodies used were: Alexa Fluor 488-, 594- and 647-conjugated antibodies. Nuclei were stained with Hoechst (1:200). Confocal images were acquired using Zeiss LSM 880 Airy inverted microscope and the ×63/1.3 LCI Plan-Neofluar objective with water immersion medium.

### Ethics statement

Informed consent was obtained from all recipients and/or donors of cells or tissues. The study involving human whole blood was covered by the ethic vote EK-BR-24/18-1 of the Sächsische Landesärztekammer. The blood was obtained from two voluntary ABO-matched donors who had not used any medicine in the past ten days. The study involving human bone marrow-derived MSCs was covered by the ethic vote EK221102004 and EK47022007 at TU Dresden. MSCs were isolated from healthy female and male donors (aged 26–37) by the Medical Faculty at the University Hospital Dresden. The study involving hiPSCs was covered by the ethic vote EK363112012 at TU Dresden. hiPSCs were generated from MACS-sorted CD34+ cells from the peripheral blood of a healthy donor (aged 20–24). The CT27 patient-derived trophoblast stem cell line used in this study had been derived from placental cytotrophoblast cells and was obtained from the RIKEN stem cell bank (RCB4936:CT27). Human placentas were obtained from healthy women with signed informed consent of the donors, and the approval of the Ethics Committee of Tohoku University School of Medicine (research license 2014-1-879).

### Statistics and reproducibility

Graphs and statistical analyses were made in GraphPad Prism and Origin 2022. Sample sizes are specified in the figure captions, where *n* refers to the number of distinct samples. For the cell viability assay, a one-way analysis of variance (ANOVA) test with a Tukey post hoc test was performed (*n* = 3, df = 4). For the blood compatibility tests, a one-way ANOVA with a Holm–Sidak post hoc test was performed (*n* = 3, df = 6). Data are expressed as mean ± s.d., with *P < = 0.05, **P < = 0.01 and ***P < = 0.001 indicating statistical significance. No statistical method was used to determine sample size, and no data were excluded from the analyses. The experiments were not randomized, and the investigators were not blinded to allocation during experiments and outcome assessment.

### Reporting summary

Further information on research design is available in the Nature Portfolio Reporting Summary linked to this article.

## Online content

Any methods, additional references, Nature Portfolio reporting summaries, source data, extended data, supplementary information, acknowledgements, peer review information; details of author contributions and competing interests; and statements of data and code availability are available at 10.1038/s41565-023-01483-3.

### Supplementary information

Supplementary Information Supplementary Notes 3.1–3.4, Figs. 1–25 and Tables 1–4.

Reporting Summary

Supplementary Table DNA sequences, cost estimation and abbreviations.

Supplementary Video 1 Extrusion of DyNAtrix from a nozzle.

Supplementary Video 2 Gel swelling in 10% FBS-supplemented DMEM for 48 h.

Supplementary Video 3 Gel protected by actin in 10% FBS-supplemented DMEM for 48 h.

Supplementary Video 4 Releasing MSCs from DyNAtrix by adding DNase I.

Supplementary Code 1 Thermodynamic prediction of the combinatorial crosslinker libraries.

Supplementary Code 2 Code for the statistical simulation in Fig. 2a

### Source data

Source Data Fig. 2 Simulation results, rheological data, source data for Fig. 2d and thermodynamic prediction.

Source Data Fig. 3 Rheological data.

Source Data Fig. 4 Rheological data.

Source Data Fig. 5 RFU value from qPCR, gel volume quantification, statistical source data of cell viabilities and immune response.

## Data Availability

Supplementary Information containing materials and methods, supplementary figures, tables, datasets and accession numbers for biological materials are available with this paper. Additional datasets and materials generated during and/or analysed during the current study are available from the corresponding author on reasonable request. The supplementary data and code supporting the findings of this study are openly available on figshare (https://figshare.com/projects/Dynamic_matrices_with_DNA-encoded_viscoelasticity_for_cell_and_organoid_culture/168281). Source data are provided with this paper.

## Code availability

A Python script for the thermodynamic calculations of CCL interactions is available as Supplementary Code 1 and at figshare (10.6084/m9.figshare.23309429). A Python script for the statistical simulation of the maximum percentage of intramolecular crosslinks as a function of CCL complexity is available as Supplementary Code 2 and at figshare (10.6084/m9.figshare.23592636).
