# Author Correction: Dynamic matrices with DNA-encoded viscoelasticity for cell and organoid culture

**DOI:** 10.1038/s41565-024-01619-z

**Published:** 2024-01-31

**Authors:** Yu-Hsuan Peng, Syuan-Ku Hsiao, Krishna Gupta, André Ruland, Günter K. Auernhammer, Manfred F. Maitz, Susanne Boye, Johanna Lattner, Claudia Gerri, Alf Honigmann, Carsten Werner, Elisha Krieg

**Affiliations:** 1 Institute for Biofunctional Polymer Materials, Leibniz Institute of Polymer Research Dresden, Dresden, Germany; 2 Center for Regenerative Therapies Dresden, Cluster of Excellence Physics of Life and Faculty of Chemistry and Food Chemistry, Technische Universität Dresden, Dresden, Germany; 3 Institute for Physical Chemistry and Polymer Physics, Polymer Interfaces, Leibniz Institute of Polymer Research Dresden, Dresden, Germany; 4 Institute for Macromolecular Chemistry, Leibniz Institute of Polymer Research Dresden, Dresden, Germany; 5 Max Planck Institute of Molecular Cell Biology and Genetics, Dresden, Germany; 6 Center for Systems Biology Dresden, Dresden, Germany

**Keywords:** Organizing materials with DNA, Molecular self-assembly, Biomaterials, Mechanical properties

Correction to: *Nature Nanotechnology* 10.1038/s41565-023-01483-3, published online 7 August 2023.

In the version of the article initially published, the authors’ first names appeared as initials rather than full names. This has now been corrected in the HTML and PDF versions of the article.

