# Supplementary material for: Dynamic matrices with DNA-encoded viscoelasticity for cell and organoid culture
Source: Nat Nanotechnol. 2023 Aug 7;18(12):1463–73. doi: 10.1038/s41565-023-01483-3 (PMC10716043; doi:10.1038/s41565-023-01483-3)
Supplement: Supplementary file 1 — Supplementary Notes 3.1–3.4, Figs. 1–25 and Tables 1–4. [file 41565_2023_1483_MOESM1_ESM.pdf]

nature nanotechnology

Article

https://doi.org/10.1038/s41565-023-01483-3

Dynamic matrices with DNA-encoded viscoelasticity for cell and organoid culture

In the format provided by the authors and unedited

nature nanotechnology

Article

https://doi.org/10.1038/s41565-023-01483-3

Dynamic matrices with DNA-encoded viscoelasticity for cell and organoid culture

In the format provided by the authors and unedited

Contents

1 Supplementary Methods 2
1.1 Polyacrylamide gel electrophoresis (PAGE) 2
1.2 Asymmetrical flow field-flow fractionation 2
1.3 Nuclear magnetic resonance (NMR) spectroscopy 2
1.4 Solid-phase peptide synthesis (SPSS) 3
1.5 General cell culture on 2D 3
1.5.1 Mesenchymal stem cells (MSCs) 3
1.5.2 Madin–Darby Canine Kidney cells (strain II, MDCK II) 3
1.5.3 Human induced pluripotent stem cells (hiPSCs) 3
1.5.4 Human trophoblast stem cells (hTSCs) 4
1.6 3D Cell culture 4
1.6.1 DyNAtrix precursors preparation 4
1.6.2 Cell encapsulation 4
1.7 Bioprinting 5
1.8 Fluorescence Live/Dead assay 5
1.9 Passaging of trophoblast organoids 5
2 Supplementary Notes 6
2.1 Benefit of a polymer backbone with high molecular weight 6
2.2 Number of DNA anchor strands per polymer molecule 6
2.3 Affine network model 6
2.4 Statistical simulations of intra- vs. inter-molecular crosslinks 7
2.5 Design of heat-activated crosslinkers (HACs) 7
3 Supplementary Figures 9
4 Supplementary Tables 28
5 Supplementary References 31

1 Supplementary Methods

1.1 Polyacrylamide gel electrophoresis (PAGE)

The binding capacity of $\mathbf{P_5}$ was determined by PAGE. native PAGE gel was prepared using 40% (w/w) acrylamide/bis-acrylamide (19:1) stock. Samples were prepared in 1x TE buffer with a final concentration of 150 mM NaCl, and 0.4 %(w/v) solutions of $\mathbf{P_5}$ (2 µM of anchor strands). The complementary strand of the anchor strand (Strand ID#1) was added at series equivalent from 0.2x~ 2x. The samples were annealed on the thermal cycler at 95°C for 2 minutes, followed by instant cooling to 4°C and hold for 3 minutes. The annealed products were loaded to a 10% native polyacrylamide gel and run at 120 V on a Mini-Cell® Novex system from Life technologies in 0.5× TBE buffer, using Consort EV265 power supply. The gel was stained with 1x SYBR™ Gold for 15 minutes and scanned on Typhoon FLA 9000 Scanners (GE Healthcare Life Sciences) using a blue LD laser (excitation at 473 nm) at 25 µm/pixel resolution. Image analysis and densitometric quantification of gel bands were performed in Fiji[1].

1.2 Asymmetrical flow field-flow fractionation

Asymmetrical flow field-flow fractionation with light scattering detection (AF4-LS) has become an important technique for gentle and detailed characterization of bio-active systems[2]. We performed the AF4 studies with an Eclipse Dualtec system (Wyatt Technology Europe, Germany). The separation takes place in a hollow fiber, a 17-cm-long polyethersulfone fiber, (Microdyn-Nadir, Germany) with 0.8 mm inner diameter, 1.3 mm outer diameter, and 10 kDa molecular weight cut-off (corresponding to an average pore diameter of 5 nm). Flows were controlled with an Agilent Technologies 1260 series isocratic pump equipped with vacuum degasser. The detection system consisted of a multiangle laser light scattering detector (DAWN HELEOS II from Wyatt Technology Europe, Germany), operating at a wavelength of 659 nm with included QELS module (at detector 99°) and an absolute refractive index detector (Optilab T-rEX, Wyatt Technology Europe GmbH, Germany), operating at a wavelength of 659 nm. All injections were performed with an autosampler (1200 series; Agilent Technologies Deutschland GmbH). The channel flow rate (Fc) was maintained at 0.35 mL/min for all Af4 operations. Samples (inject load: ~50-100 µg) were injected during the focusing/relaxation step within 5 min. The focus flow was set to 0.85 mL/min. During the elution step, the cross-flow rate (Fx) was optimized by an exponential cross-flow gradient of 0.85 - 0.03 mL/min in 30 min. 10 mM TRIS buffer and 1 mM EDTA (pH 8.0) were used as eluent for all measurements. Collecting and processing of detector data were made by the Astra software, version 6.1.7 (Wyatt Technology, USA). The molar mass dependence of elution time was fitted with Berry (first-degree exponential).

1.3 Nuclear magnetic resonance (NMR) spectroscopy

NMR samples were prepared by diluting 100 µL of the unpurified product in 700 µL of D_2O. ^1H NMR spectra were recorded at 30-32°C on a 500 MHz spectrometer (Bruker) with 2 seconds acquisition time and 32 transients. Chemical shifts (δ) are reported in parts per million (ppm) downfield from tetramethyl silane (TMS). ^1H NMR shifts are referenced to the residual hydrogen peak of D_2O (4.79 ppm).

1.4 Solid-phase peptide synthesis (SPSS)

All SPPS chemicals were purchased from IRIS Biotech GmbH. RGD peptides (sequence: G(acryl-K)GGGRGDSP) were synthesized on a Liberty Blue HT12™ automatic and microwave-assisted peptide synthesizer (CEM GmbH) using a Rink Amide resin and a 9-fluorenylmethoxycarbonyl protection strategy. Amino acid activation was achieved by N, N'-diisopropyl carbodiimide, and ethyl cyanohydroxyiminoacetate. Acetylation of the N-terminus was performed via incubating the resin-bound peptides in acetic anhydride for 2 h. The reaction mixture was continuously stirred and vigorously saturated by bubbled nitrogen to prevent oxidation reactions and afterward washed three times with N, N-dimethylformamide. Deprotection of the amino acid side chains and cleavage from the resin was accomplished using a mixture of trifluoroacetic acid (ϕ_i = 87.5%), phenol (ϕ_i = 5%), triisopropylsilane (ϕ_i = 2.5%) and MilliQ-water (ϕ_i = 5%) for 3 h at room temperature. The crude peptide was precipitated in anhydrous diethyl ether, collected by vacuum filtration, and dried under nitrogen flow. Further purification of the peptides was realized by high-performance liquid chromatography (Agilent 1200, Agilent Technologies) on a preparative C18 column (10 µm particle size, 100 Å pore size, 250 x 30 mm, Phenomenex Ltd). A linear gradient of MilliQ-water/acetonitrile and trifluoroacetic acid (ϕ_i = 0.1%) was used as the mobile phase. Finally, the concentrated peptide-containing solution was lyophilized (Alpha 2-4 LD plus freeze-dryer, Martin Christ) and the obtained product was stored at -20°C afterward until further usage.

1.5 General cell culture on 2D

1.5.1 Mesenchymal stem cells (MSCs)

Mesenchymal stem cells (MSCs) were cultured in Dulbecco's Modified Eagle's medium (DMEM) GlutaMAX™ (Gibco, Cat. #21885025) supplemented with 10% Fetal Bovine Serum (FBS) (Sigma Aldrich, Cat. # F7524) and 100U Penicillin and 0.1 mg Streptomycin (Sigma Aldrich, Cat. #P4333) at 37°C and 5% CO_2 in a humidified incubator. The cells were grown until 80%-90% confluence in T75 flask before detachment with 0.25% trypsin-EDTA solution.

1.5.2 Madin–Darby Canine Kidney cells (strain II, MDCK II)

Auto-fluorescent Madin–Darby Canine Kidney cells (strain II, MDCK II) were provided by Alf Honigmann's group (MPI-CBG). These cells express E-cadherin-mNeonGreen and podocalyxin-mScarlet. The cells were cultured in minimum essential medium (MEM) with GlutaMAX™ Supplement (Gibco, Cat. #11140050), 1%MEM Non-Essential Amino Acids Solution (100X) (Gibco, Cat. #41090028), 1mM Sodium Pyruvate (Gibco, Cat. #11360070), 5% FBS (Sigma Aldrich, Cat. #F7524), and 100U Penicillin and 0.1 mg Streptomycin (Sigma Aldrich, Cat. #P4333) at 37°C and 5% CO_2 in a humidified incubator. The cells were grown until 80%-90% confluence in T75/T25 flask before detachment with 0.25% trypsin-EDTA solution.

1.5.3 Human induced pluripotent stem cells (hiPSCs)

Human induced pluripotent stem cells (hiPSCs) were cultured in mTeSR™1 (StemCell, Cat. # 85857) supplemented with 100U Penicillin and 0.1 mg Streptomycin (Sigma Aldrich, Cat. #P4333) on a 6-well plate coated with Matrigel (Corning, Cat. #354277). The cells were grown until 90% confluence and then detached by ReLeSR (StemCell, cat # 05872). The cell suspension was split at 1:4 or 1:6 ratio and seeded onto a new coated plate. For encapsulation, the obtained cell suspension was transferred to a low-

adhesion 6-well plate to form cell aggregates in mTeSR™1 supplemented with 8 µM ROCK inhibitor Y-27632 (StemCell, cat # 72304) overnight.

1.5.4 Human trophoblast stem cells (hTSCs)

Human trophoblast stem cells (hTSCs) were cultured as described previously[3]. In brief, a 6-well plate was coated with 5 µg/mL Col IV at 37°C for at least one hour. The cells were seeded and cultured in the TSC medium [DMEM/F12 supplemented with 0.1 mM 2-mercaptoethanol, 0.2% FBS, 0.5% Penicillin-Streptomycin, 0.3% BSA, 1% ITS-X supplement, 1.5 µg/mL L-ascorbic acid, 50 ng/mL EGF, 2 µM CHIR99021, 0.5 µM A83-01, 1 µM SB431542, 0.8 mM VPA and 5 µM Y27632] at 37°C and 5% CO2 in a humidified incubator. The TSC medium was replaced every two days. The cells were grown until 80% confluence before detachment with TrypLE (Thermo Fisher Scientific, Cat#12604031).

1.6 3D Cell culture

1.6.1 DyNAtrix precursors preparation

All materials were dissolved in water and stored at -20°C for cell culture experiments. For MSC and hiPSC cultures, two precursors were prepared at a final concentration of 1% (w/v) $\mathbf{P_5^{RGD}}$ with: (1) 37.5 µM forward splint (Strand ID #6a) and 75 µM blocking strand (Strand ID #15a), and (2) 37.5 µM reverse splint strands (Strand ID #6b) and 75 µM blocking strand (Strand ID #15b). As for MDCKII and hTSC cultures, two precursors were prepared at a final concentration of 1% (w/v) $\mathbf{P_{10}^{RGD}}$ with: (1) 75 µM forward splint (Strand ID #6a) and 150 µM blocking strand (Strand ID #15a), and (2) 75 µM reverse splint strands (Strand ID #6b) and 150 µM blocking strand (Strand ID #15b). Concentrated DMEM was added to the precursors to reach 1x final concentration. The two precursor solutions were equilibrated at 4°C overnight to allow the blocking strands to quantitatively bind to the splint strands.

1.6.2 Cell encapsulation

Cell suspension was mixed with the first precursor (1). Subsequently, it was mixed with the second precursor (2) on ice to reach a final cell density of:

- MSCs: 1 x 10^6 cells/mL
- MDCKII: 4 x 10^5 cells/mL
- hiPSCs: 5 x 10^3 aggregates/mL
- hTSCs: 4 x 10^5 cells/mL

5-8 µL/well of the cell-DyNAtrix mixture were subjected onto 384-well plates or 6-well plates containing micro-well inserts (ibidi, Cat. 80409). The samples were incubated at 37°C for 20-40 minutes to trigger the heat-activated gelation. After gelation, the well was filled up with individual culture medium:

- MSCs: Dulbecco's Modified Eagle's medium (DMEM) GlutaMAX™ (Gibco, Cat. #21885025) supplemented with 10% Fetal Bovine Serum (FBS) (Sigma Aldrich, Cat. # F7524) and 100U Penicillin and 0.1 mg Streptomycin (Sigma Aldrich, Cat. #P4333).
- MDCKII: Minimum essential medium (MEM) with GlutaMAX™ Supplement (Gibco, Cat. #11140050), 1X MEM Non-Essential Amino Acids Solution (Gibco, Cat. #41090028), 1mM Sodium Pyruvate (Gibco, Cat. #11360070), 5% FBS (Sigma Aldrich, Cat. # F7524), and 100U Penicillin and 0.1 mg Streptomycin (Sigma Aldrich, Cat. #P4333)

- hiPSCs: mTeSR™1 supplemented with 8 µM ROCK inhibitor Y-27632 in the first 24 hours, and fresh mTeSR™1 without ROCK inhibitor for the rest of the culture.
- hTSCs: organoid medium [DMEM/F12 supplemented with 1x N2 supplement, 1x Glutamax, 1x B27 supplement without vitamine A, 100 µg/mL Primocin, 1.25 mM N-acetyl-L-cysteine, 1.5 µM CHIR99021, 50 ng/mL human EGF, 80 ng/mL human R-spondin-1, 100 ng/mL human FGF-2, human 50 ng/mL HGF, 0.5 µM A83-01, 2.5 µM Prostaglandin E2, and 2 µM Y27632].

When serum-containing medium was used, 100µg/mL of rabbit skeletal muscle actin was added to suppress nuclease activity. The embedded cells were cultured at 37°C in a humidified incubator equilibrated with 5% CO_2.

1.7 Bioprinting

MDCK II cells were encapsulated in 400 µL *DyNAtrix* (1 %(w/v) $\mathbf{P_5^{RGD}}$ + CCL-64 with blocking strands) following the protocol 2.15. The cell-laden hydrogel was transferred directly into a 1 ml Luer-Lock syringe (Omnifix®-F Solo, Cat. # 916700) with a 30-guage dispensing tip (Vieweg, Cat. # 500908). The syringe was centrifuged at 300 Xg for 3 min to remove air bubbles. The cell-laden gel was printed on a glass-bottom 6-well plate on a BioScaffolder BS5.1 (GeSiM) at a speed of 2 mm/s and an extrusion rate of 40 µm/s for 2 layers with 0.12 mm layer height. One hour after printing, the constructs were stained in 200 µL medium containing 3 µM calcein-AM, 0.75 µM Draq7, and 2 drops/mL NucBlue for 30 minutes. The images were acquired on Opera Phenix Plus High-Content Screening System at 10x magnification.

1.8 Fluorescence Live/Dead assay

For hiPSCs, MDCK II, and trophoblast organoids, *DyNAtrix* was degraded by 160U/ml DNase I in 250 µL medium at 37°C, 5% CO_2 before live/dead staining. Matrigel was degraded in 250 µL cell recovery solution (Corning, Cat. # 354253). After degradation, cells were washed with medium once and incubated in staining medium [3 µM calcein-AM, 0.75 µM Draq7, and 2 drops/mL NucBlue (invitrogen, Cat # R37605)] for 30 minutes at 37°C, 5% CO_2. The confocal images were acquired either at 20x magnification on Andor Dragonfly Confocal Microscope or at 30x magnification on Andor Revolution WD Confocal Microscope (Oxford instrument).

1.9 Passaging of trophoblast organoids

We followed and adapted a recent protocol established by the Turco Lab[4]. In brief, the gels were collected to an Eppendorf tube and degraded on day 7 of culture. We added 150 µL DMEM/F12 medium to Matrigel (Corning, Cat. #356231) samples and pipetted them for 400 times through a small-bore tip to break up the gels. For *DyNAtrix*, we added 160U DNase I in 150 µL medium and incubated them for 40 minutes to degrade the gels. Matrigel and *DyNAtrix* were added with 1 mL DMEM/F12 and centrifuge at 600g for 6 min at RT. After removing the supernatant, all samples were added with 500 µL of pre-warmed Accutase and incubated for 5-6 min at 37°C. After incubation, the samples were washed once with 1 mL DMEM/F12. We suspended the organoids in 150 µL DMEM/F12 medium and pipetted for 80 times to break up the organoids. Finally, the samples were centrifuged at 600g for 6 min and re-suspended in 20 µL DMEM/F12 medium. The cell suspension was embedded in the gels at a final 1:4 splitting ratio.

2 Supplementary Notes

2.1 Benefit of a polymer backbone with high molecular weight

Unless there is significant entanglement (i.e., at high polymer concentration and high molecular weight), a polymer molecule can only contribute to the elasticity of the material, if it has more than 2 crosslinks that connect it to the network[5–7]. If the weight of the polymer backbone was only a few kilodalton, even a small number of DNA strands per backbone would represent the majority of the material by mass, since each DNA strand has a molecular weight of several kilodalton (~7kDa for each anchor strand in DyNAtrix). In fact, in most DNA-crosslinked polymer hydrogels the DNA crosslinkers represent the majority of the dry weight of the material[8,9]. In contrast, DyNAtrix has an ultra-high molecular weight backbone (~3 MDa; Tables S2 and S3), achieved by low initiator concentration and stringent exclusion of oxygen during polymerization. In $\mathbf{P_1}$, $\mathbf{P_5}$, and $\mathbf{P_{10}}$ the calculated weight fraction of DNA is only 1.0%, 4.8% and 9%, respectively. The DNA handles can thus represent only a minor fraction of the material's dry weight while efficiently contributing to the material's elastic properties.

2.2 Number of DNA anchor strands per polymer molecule

To estimate the number of anchor strands, we used the equation

$$M_P = M_A \cdot N_A + M_{AA} \cdot N_{AA} + M_{DNA} \cdot N_{DNA}$$

where M_P is the number average molecular weight of the polymer chain (Table S2); M_A, M_{AA}, and M_{DNA} are the molecular weights of acrylate (A), acrylamide (AA), and acrylamide-labeled DNA groups, respectively; N_A, N_{AA}, and N_{DNA} are the numbers of corresponding monomers in the polymer chain.

As $M_A \cdot N_A$ << $M_{AA} \cdot N_{AA}$ for all polymer derivatives, we used the simplified equation

$$M_P \approx M_{AA} \cdot N_{AA} + M_{DNA} \cdot N_{DNA}$$

The monomers ratio r = N_{AA}/N_{DNA} in the synthesis is ~$7 \cdot 10^3$, $1.4 \cdot 10^3$, and $7 \cdot 10^2$ for $\mathbf{P_1}$, $\mathbf{P_5}$, and $\mathbf{P_{10}}$, respectively. Since ~75% of DNA anchor strands are both included in the polymer and accessible for binding (Figure S2), r was corrected to be ~$1 \cdot 10^4$, $2 \cdot 10^3$, and $1 \cdot 10^3$ for $\mathbf{P_1}$, $\mathbf{P_5}$, and $\mathbf{P_{10}}$, respectively. Replacing N_{AA} and rearranging the equation we obtain:

$$N_{DNA} \approx \frac{M_P}{r \cdot M_{AA} + M_{DNA}}$$

2.3 Affine network model

According to the affine network model, the elastic modulus, G', is directly proportional to the density of effective (i.e. *inter*-molecular) crosslinks, v_e:[5]

$$G' = A \cdot v_e \cdot R \cdot T$$

where R is the ideal gas constant, T is the temperature in Kelvin degree, and A is a constant that equals to 1 for affine networks. The equation was used to derive v_e values. Crosslinking efficiencies (CE) were

calculated as CE = v_e / $v_{e,\max}$, where $v_{e,\max}$ is the maximally possible crosslinker density at a given concentration, i.e. the total concentration of splint strands in the systems.

2.4 Statistical simulations of intra- vs. inter-molecular crosslinks

Description. Statistical simulations were carried out with a custom script that was written in Python 3. The script randomly selects N_a crosslinker strands from a crosslinker library of size N_x. N_a denotes the available number of anchor sites per polymer chain. The script quantifies the fraction of strands that would find a perfectly complementary binding partner on the same polymer chain to determine the maximal percentage of intra-molecular crosslinks. Na and Nx are set to [1, 2, 4, 8, 16, 32, 64, 128, 256, 512], the script systematically scans all Na vs Nx combinations, and this scan is repeated at least 100 times to create an average. The results are then exported in the form of a heatmap plot.

Limitations. While the statistical simulation predicts an upper limit for intra-molecular bonds, it does not account for kinetic or certain topological effects[10]. For instance, increasing library complexity from 64 to 256 splint pairs did not further increase stiffness. We suspect that the marginal benefit of using *CCL-256* is likely offset by its slower binding kinetics, since overlap domains of polymer-bound crosslinker strands become increasingly unlikely to find and capture their zero-mismatch binding partners in very large libraries. We also note that CCLs are expected to improve crosslinking efficiency only when the solid content of the gel is low. This is because at high concentrations and under equilibrium conditions inter-molecular bonds can outcompete intra-molecular loops. Moreover, intra-molecular bonds can also mechanically interlock, thereby contributing to the network's elasticity[10].

2.5 Design of heat-activated crosslinkers (HACs)

The addition of blocking strands (**B**) converts ordinary crosslinker splints (**S**) into HACs (see scheme below). **B** was designed to bind a large part of the crosslinker overlap domain (**c**/**c'**) to prevent premature crosslinking at low temperature. The length of the binding sequence (**b**/**b'**) was chosen to be 10 nt. This domain length was expected to be stable at 4°C, while at 37°C (close to its predicted melting temperature, T_m ~ 40°C) **S** and **B** were expected to frequently dissociate and re-associate. No more than 2 consecutive bases were allowed to remain unpaired on ***c***, since longer single-stranded domains could potentially act as toeholds for a strand displacement pathway that would lead to premature crosslinking at low temperature. In order to provide additional thermodynamic driving force for the crosslinking, **b** was flanked by two 2-nt single-stranded overhangs ($\mathbf{f_1}$ and $\mathbf{f_2}$). Analogously, **b'** was flanked by two 2-nt domains ($\mathbf{f_1}'$ and $\mathbf{f_2}'$) that were complementary to the flanking domains in **b**. The purpose of the **f** domains was to add a thermodynamically favorable bias to the crosslinking reaction: upon their release from **S**, two complementary **B** strands can form a 14-nt dimer that is thermodynamically stable, thereby removing the blocking strands from the equilibrium. Specific blocking strand sequences are listed in Table S1.

We note that in absence of blocking strands DyNAtrix is relatively stiff at 4°C and drastically softens upon heating to 37°C. This finding suggests that during initial mixing numerous *base-mismatched* crosslinks are rapidly formed, yet they are labile and dissociate upon heating, leaving behind a network with few effective crosslinks (Main text Figure 4c, green trace).

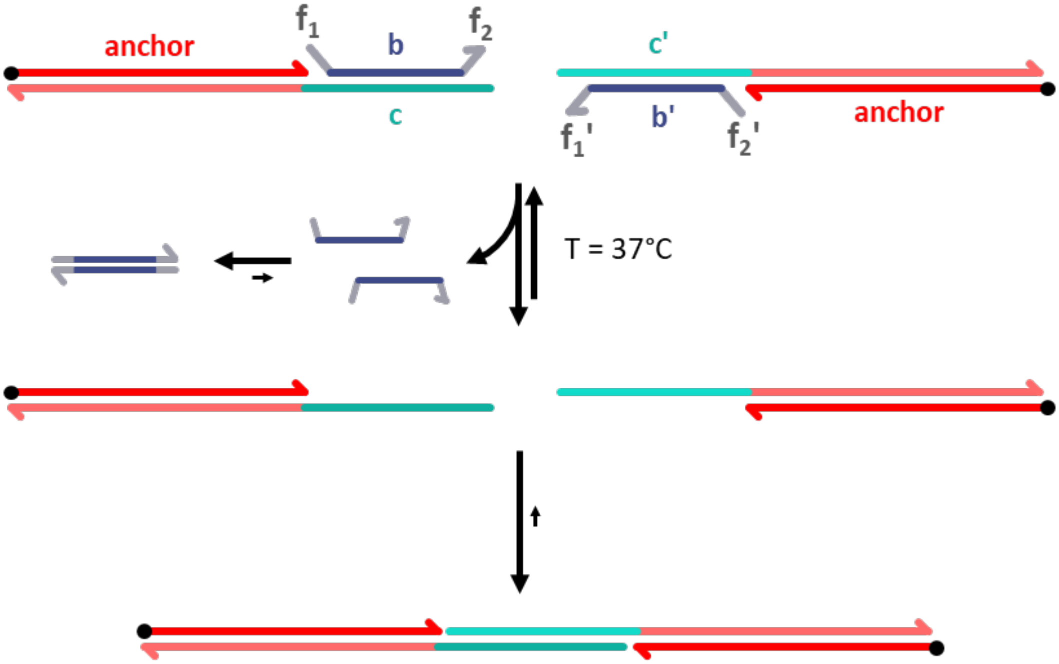
anchor
f1
b
f2
c
c'
b'
f1'
f2'
anchor
T = 37°C

3 Supplementary Figures

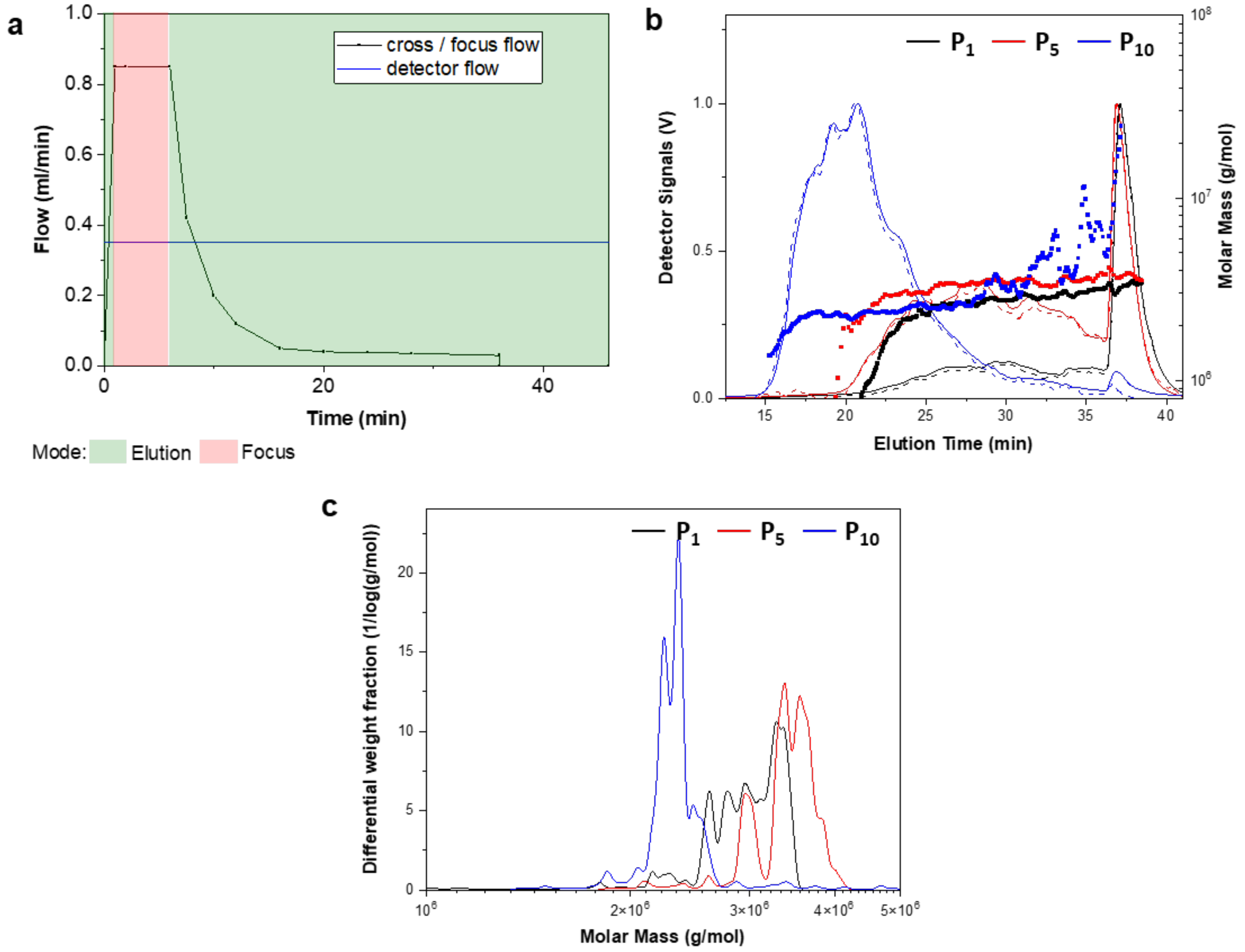

Figure S 1. Asymmetrical flow field-flow fractionation with light scattering (AF4-LS) of ***P_1, P_5, and P_{10}****. a) Optimized flow profile for AF4 separation, b) Fractograms, detector signals (solid line: multi-angle light scattering, dashed line: refractive index) and c) molar mass distributions.*

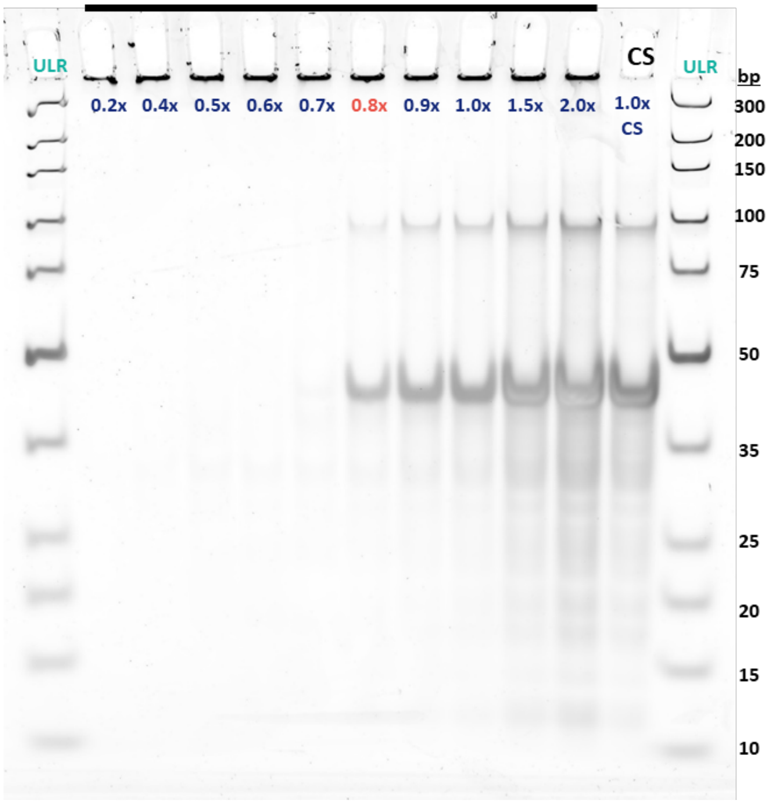

Figure S 2. DNA binding efficiency test to quantify the number of accessible anchor strands per polymer chain. Taking $\boldsymbol{P_5}$ *as an example, 0.4 % (w/v) polymer, which contains a maximum concentration of 2 µM anchor strands, was mixed with the complementary strand (CS) at ratios ranging from 0.2x to 2.0x. Complementary strands at concentrations below 0.8x did not appear as distinct bands on the gel, indicating that they were quantitatively bound to the polymer. 0.8x of complementary strands saturated the anchor strands and show clear bands of excessive CS on the gel. Thus, the concentration of available anchor strands was determined to be approximately 1.5 µM.*

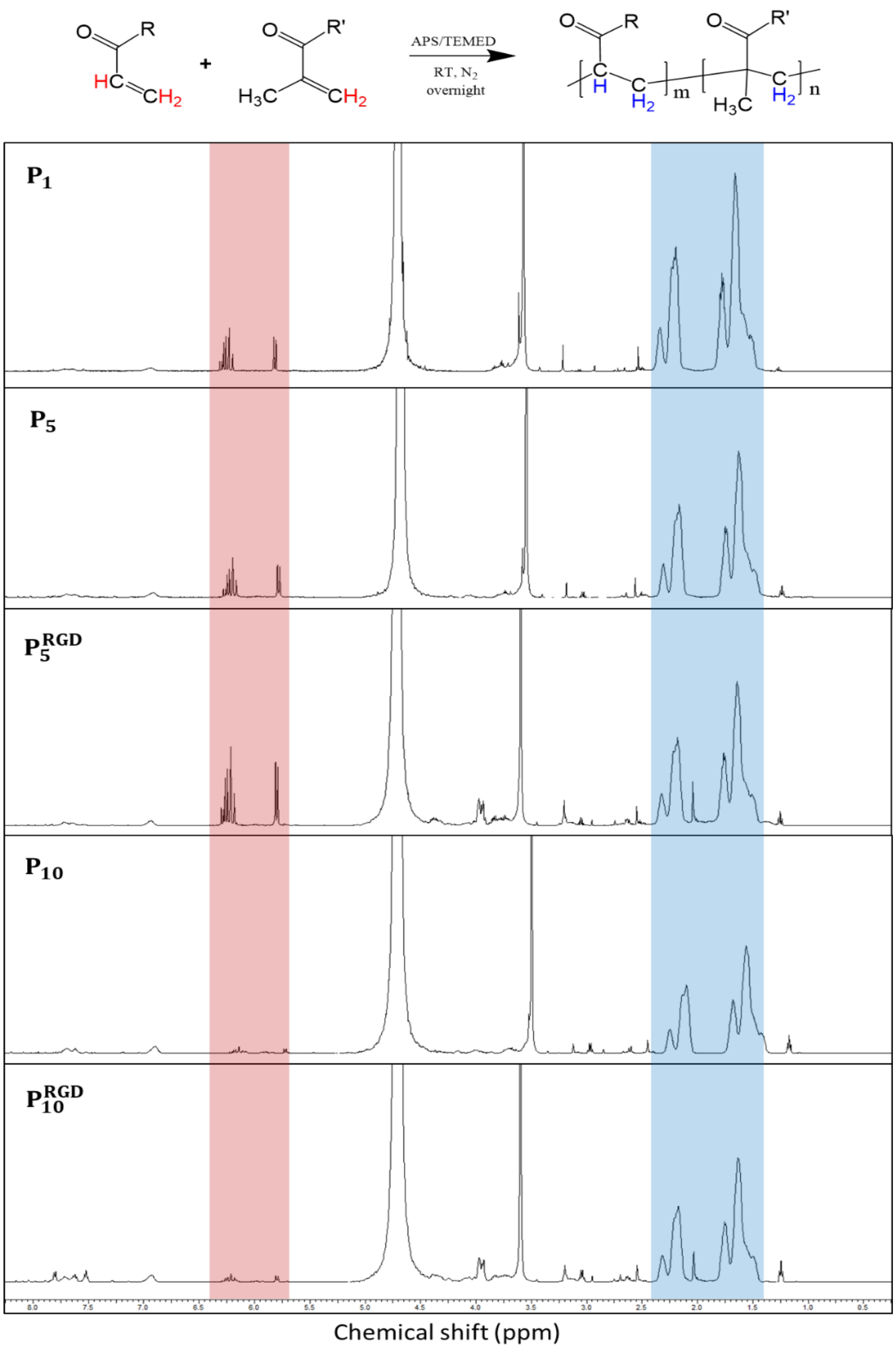

*Figure S 3. ^{1}H-NMR spectra of **P_1**, **P_5**, **P_5^{RGD}**, **P_{10}**, and **P_{10}^{RGD}** in D_2O after completed reaction and prior to methanol precipitation purification. The conversion of the reaction was calculated by quantifying free residual acrylamide monomer protons (δ~5.7-6.4; red) vs. polymer backbone protons (δ~1.4-2.4; blue). Conversions are 96%, 94%, 90%, 99%, and 98% for **P_1**, **P_5**, **P_5^{RGD}**, **P_{10}**, and **P_{10}^{RGD}**, respectively. R = -NH_2 , -OH or -NH-peptides; R' = -NH-oligonucleotides.*

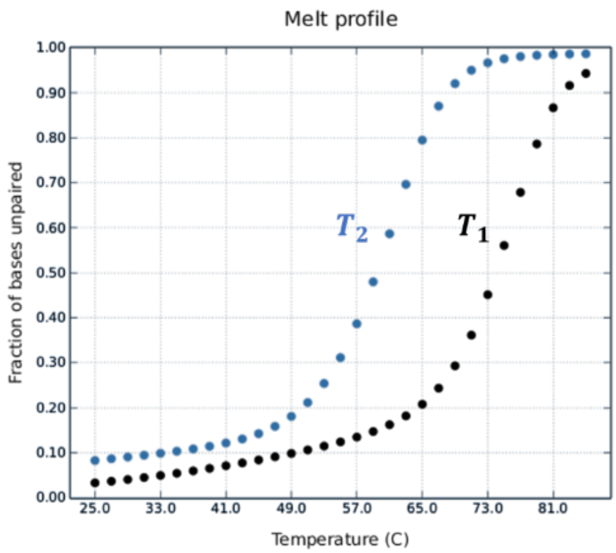

Figure S 4. Predicted melting profile of the 22-nt anchor domain (black) and a 14-nt overlap domain (blue) of dual splint crosslinkers. Splints are predicted bind to anchor strands at a higher melting temperature (T_1) and then pair to a complementary splint at a lower melting temperature (T_2). The melting profiles were simulated in NUPACK[11].

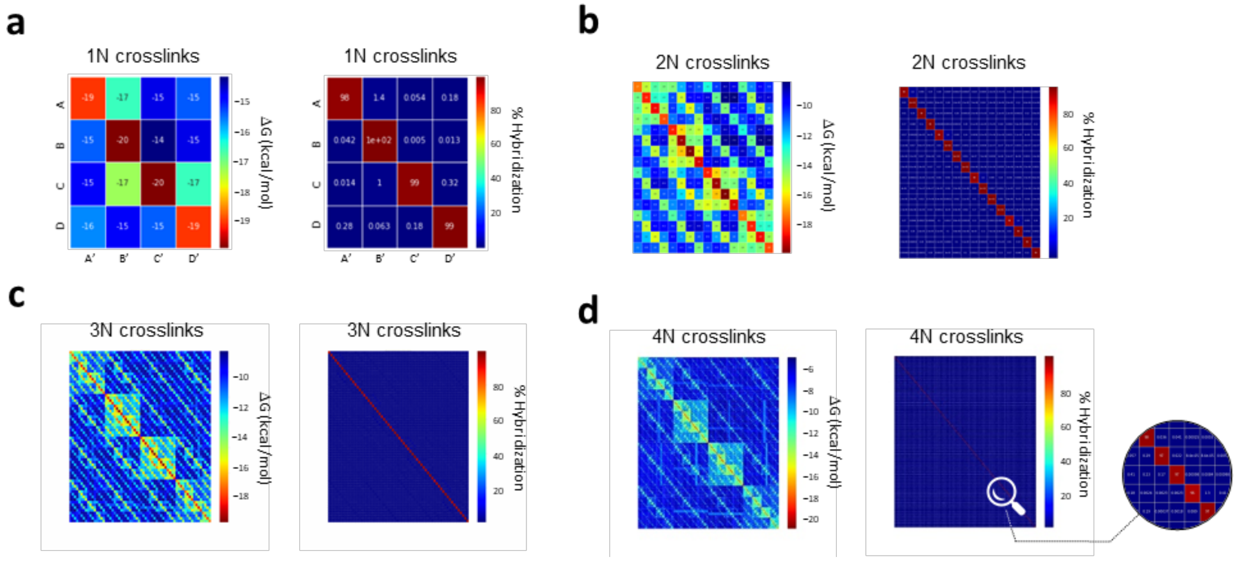

Figure S5. Predicted minimum free energy (left) and the normalized Boltzmann distribution (right) of the combinatorial crosslinker libraries with 1 – 4 ambiguous bases. The simulation was carried out using NUPACK[11]. High resolution images are available on Figshare (https://doi.org/10.6084/m9.figshare.23309429).

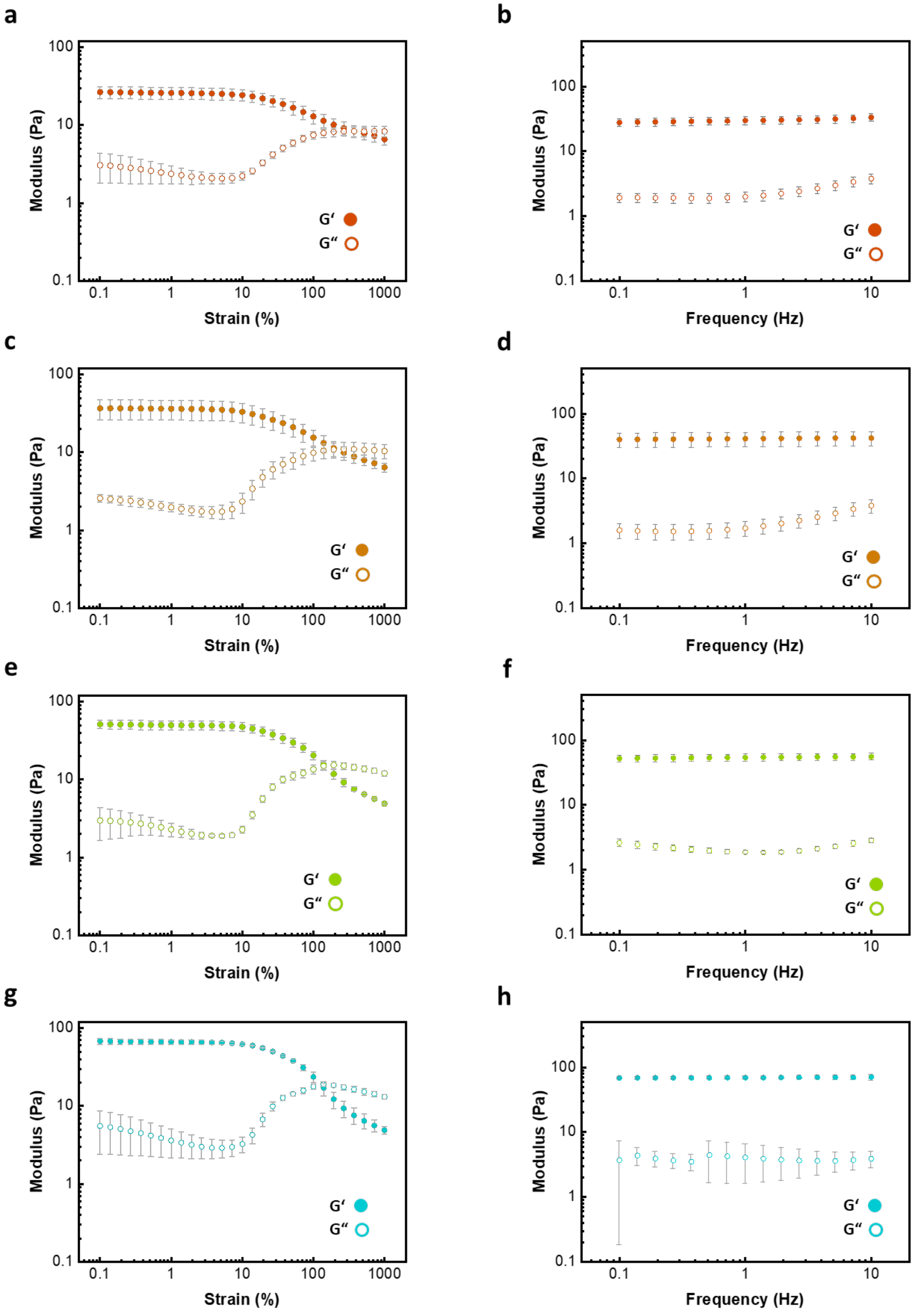
a
b
c
d
e
f
g
h
Modulus (Pa)
Strain (%)
Frequency (Hz)
G'
G"
0.1
1
10
100
1000

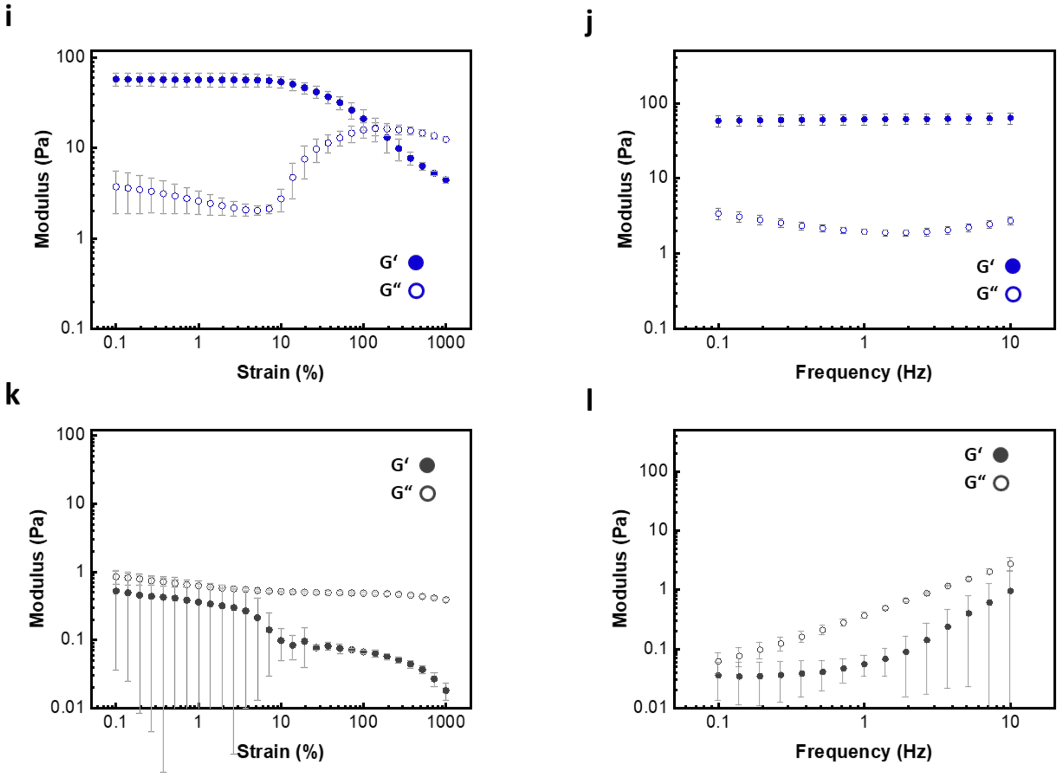

Figure S 6. Amplitude sweeps (left) and frequency sweeps (right) of 1% (w/v) $\boldsymbol{P_5}$ *hydrogels crosslinked by (a,b) CCL-1; (c,d) CCL-4; (e,f) CCL-16; (g,h) CCL-64; (i,j) CCL-256; or (k,j) without CCL. All data are shown as mean ± S.D. (n=3). All samples were measured at 37℃.*

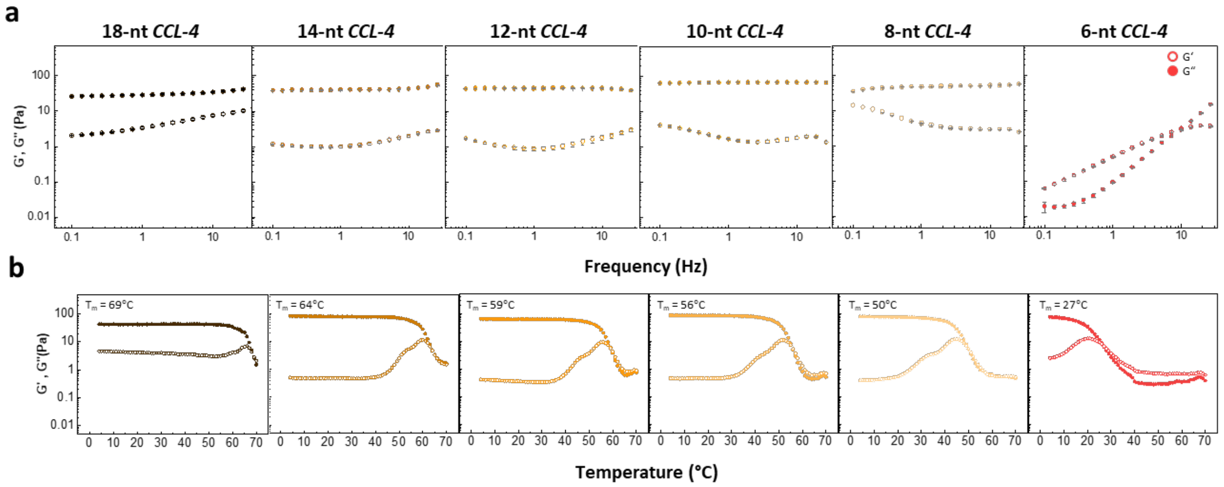

Figure S 7. (a) Frequency sweeps (0.1–30 Hz) of SRC-crosslinked $\boldsymbol{P_5}$ *(1% (w/v)) at 37°C. SRCs represent CCL-4 with overlap domains ranging from 6 to 18 nt. All gels exhibit gel-like mechanical properties (G'>>G''), except for the 6-nt CCL-4, which is solid-like at 37°C only at high frequencies (>10 Hz). Data are shown as mean ± S.D. from one hysteresis measurement. (b) Temperature-dependent curves (4–70°C) of the respective samples in (a). Below their melting temperature, all gels exhibit a plateau region with comparable storage moduli. Melting temperatures,* T_m*, defined as the crossover temperature for G' and G'', are significantly above 37°C for SRCs between 8 and 18 nt.*

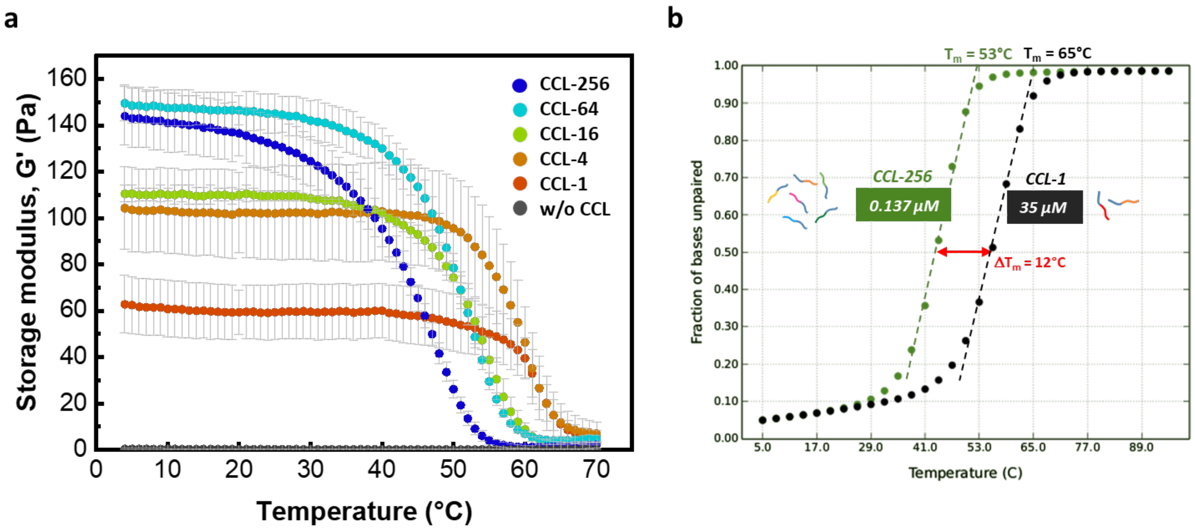

Figure S 8. (a) Temperature sweep of 1% (w/v) ***P_5*** *hydrogels crosslinked by different CCLs. The samples were measured at 10% strain and 1.6Hz in an oscillatory rheometer. Data points are shown as mean ± S.D. (n=2). (b) NUPACK calculation for predicting the melting temperature (T_m) of different splint concentrations corresponding to the actual concentration of distinct splint pairs in CCL-1 (35 µM) and CCL-256 (0.137 µM).*

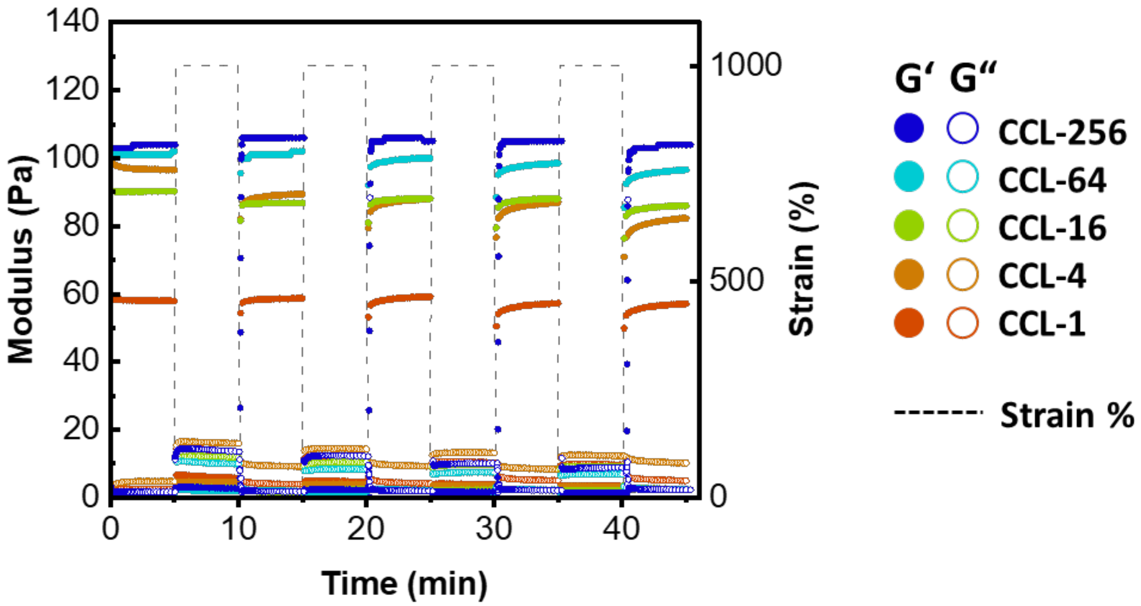

Figure S 9. Rapid self-healing tests of 1% (w/v) ***P_5*** *hydrogels crosslinked by different CCLs. The samples were sheared at 1000% (disruption) and 10% (recovery) strain at 1.6Hz, 37℃ for 5 repetitive cycles.*

Crosslinking CCL-grafted polymers

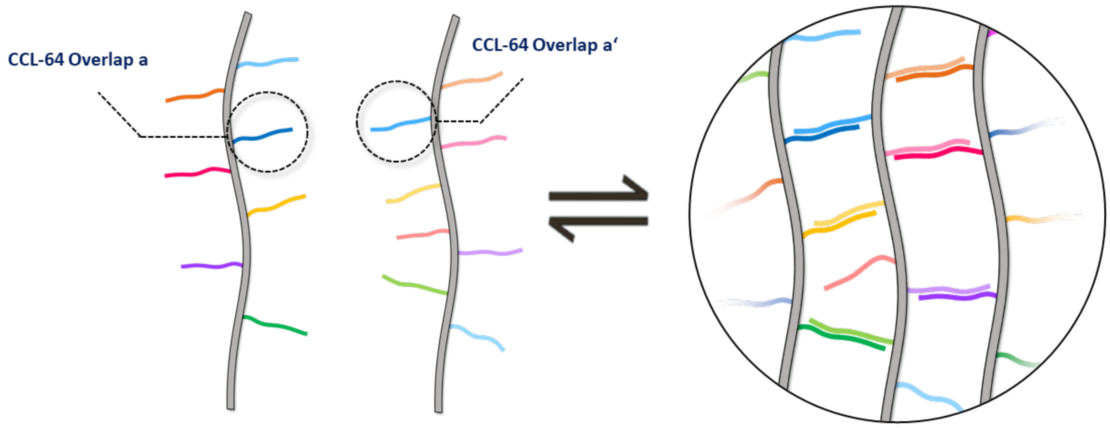

Figure S 10. The scheme of crosslinking two complementary CCL-grafted polymers. To reduce DNA content, an overlap domain can be directly grafted onto the polymer backbone. When the two polymers are well mixed and thermally annealed they form stable supramolecular networks.

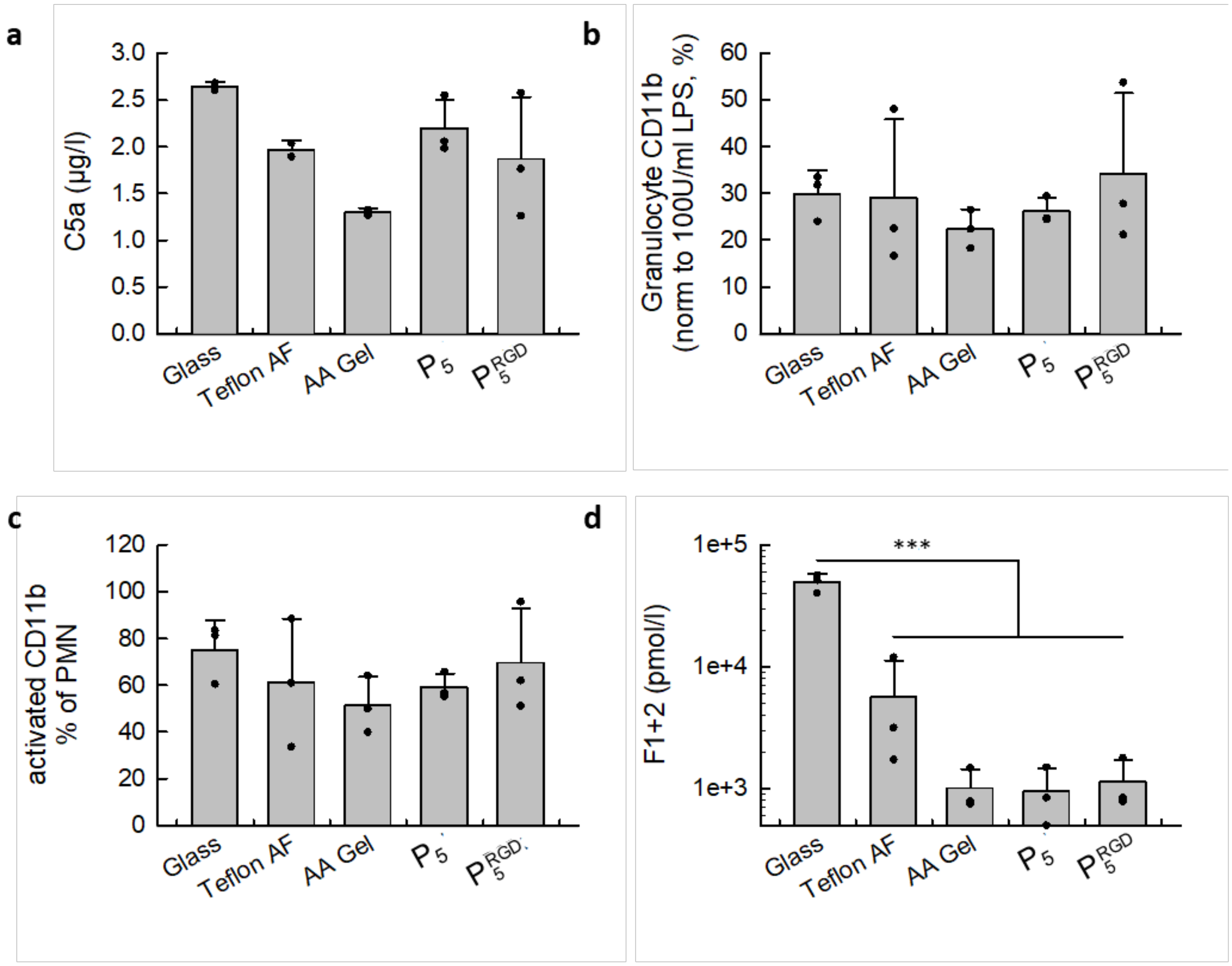

*Figure S 11. Innate immune response and hemocompatibility tests. 1% (w/v) $\mathbf{P_5}$ and $\mathbf{P_5^{RGD}}$ hydrogels crosslinked with CCL-64 were incubated in whole human blood. a) Markers for complement activation (C5a), b) granulocytes CD11b, c) activated CD11b as fraction of granulocytes, and d) hemostasis marker prothrombin fragment 1+2 (F1+2). All data are shown as mean ± S.D. (n=3).Statistical analysis was performed using one-way ANOVA followed by Holm-Sidak post-hoc test. (***, $p < 0.001$).*

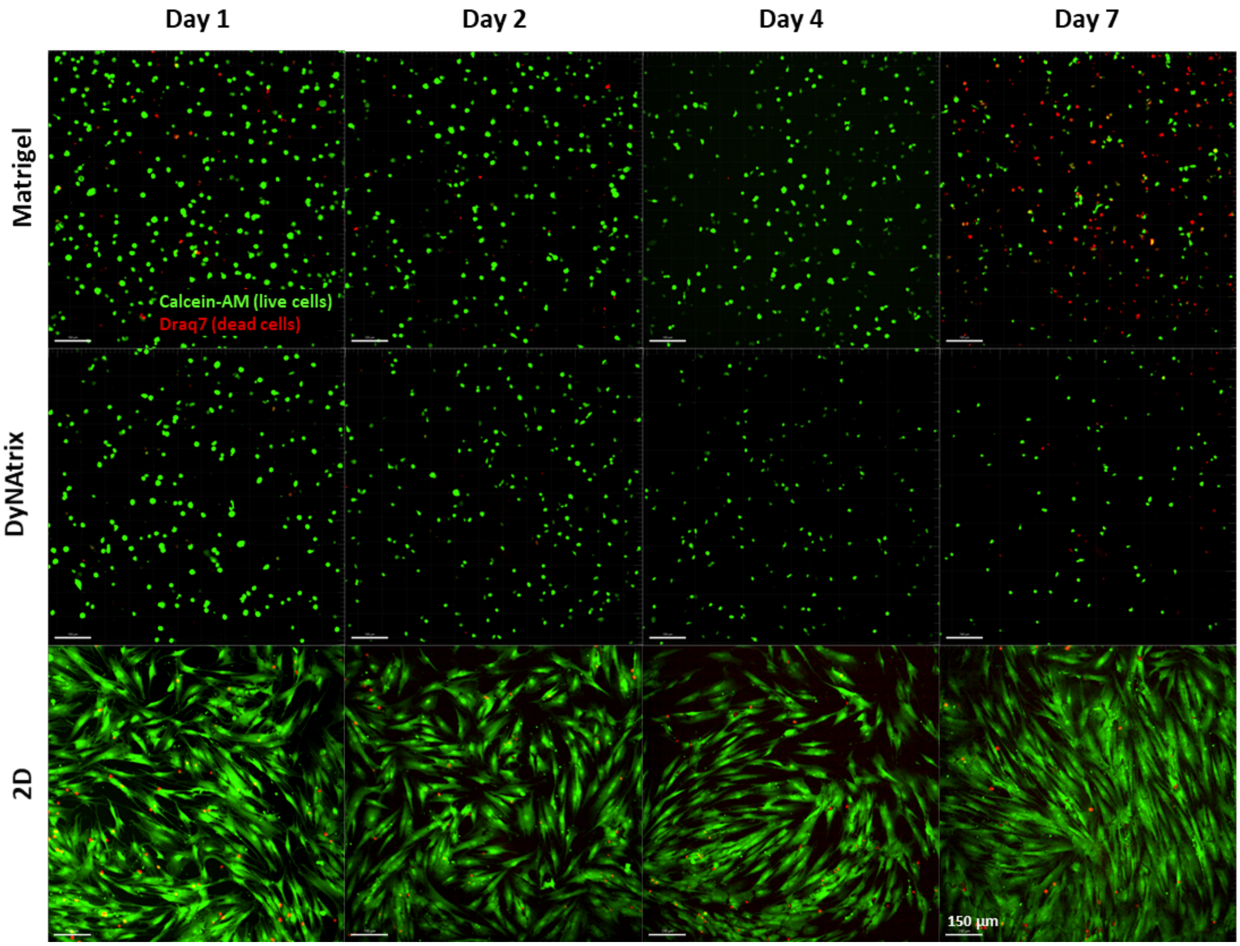

Figure S 12. hMSCs viability tests with calcein-AM (live cells) and Draq7 (dead cells). The cells were cultured in Matrigel, DyNAtrix (1% (w/v) $\boldsymbol{P_5^{RGD}}$ + CCL-64), and a 2D cell culture dish. Variations in cell density between days 1 and 2 are due to initial swelling of the gels. The viability is calculated using the ratio of total live cell divided by the sum of live and dead cells. Scale bar = 150 µm.

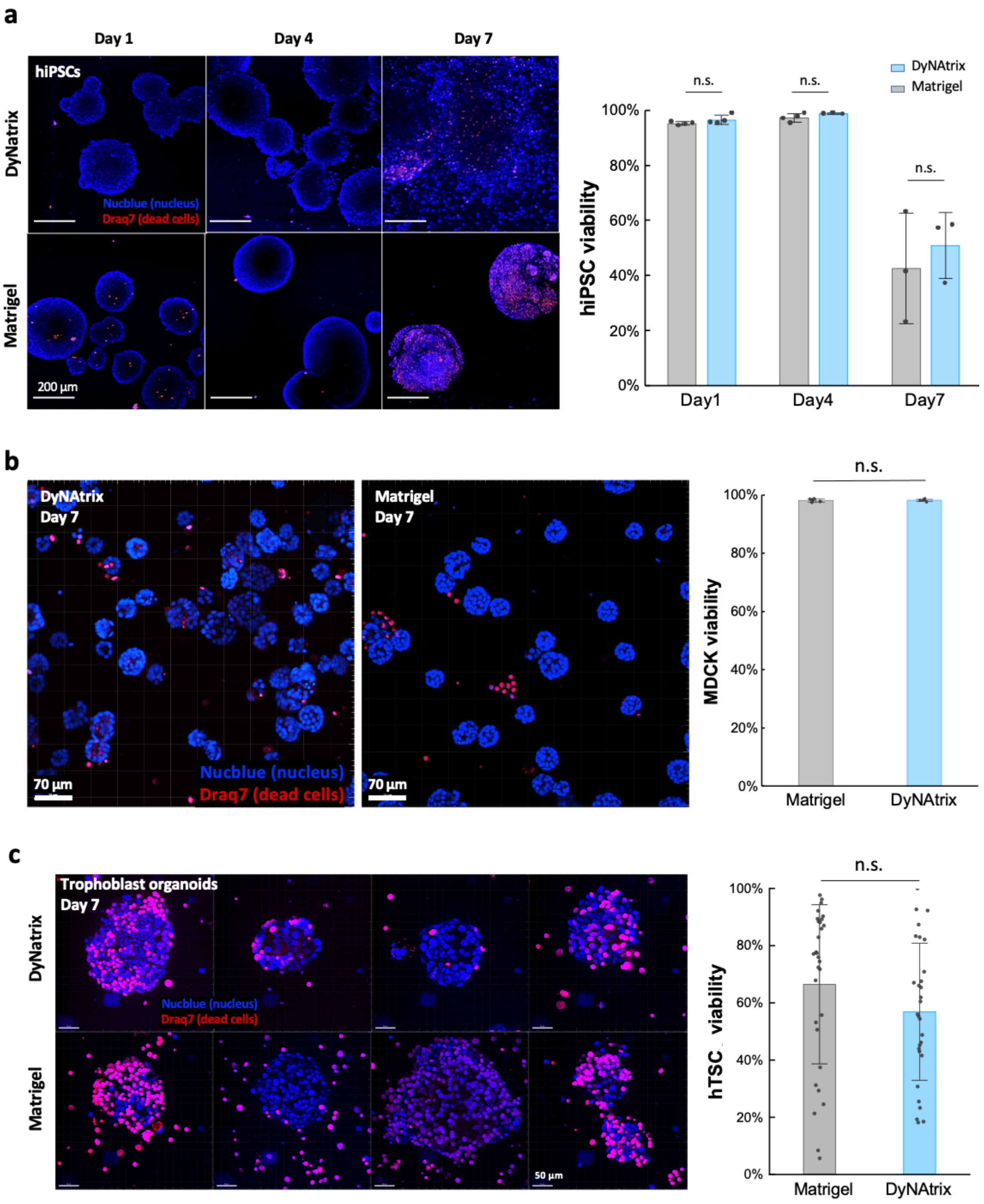

Figure S 13. Viability tests of hiPSC (a), MDCK cells (b), and trophoblast organoids (c) stained with Draq7 (dead cells) and NucBlue (nuclei). a) hiPSCs were cultured in DyNAtrix (1% (w/v) $\mathbf{P_5}^{RGD}$ + CCL-64) and Matrigel. Viability was high (>97%) for DyNAtrix and Matrigel on days 1 and 4. On day 7 increased cell death was observed in both matrices, likely because the size and density of the cysts became too large for sufficient nutrient uptake. Data are shown as mean ± S.D. (n=4 on day 1 and day 4; n=3 on day 7 due to gel loss during the staining procedure). Statistical analysis was evaluated with a two-way ANOVA with Tukey post-hoc test. b) MDCK cells were cultured in DyNAtrix (1% (w/v) $\mathbf{P_{10}}^{RGD}$ + CCL-64) and Matrigel. Data are shown as mean ± S.D. (n=5). Statistical analysis was evaluated with an unpaired two-sample t-test. c) hTSCs were cultured in DyNAtrix (1% (w/v) $\mathbf{P_{10}}^{RGD}$ + CCL-64) vs. Matrigel and grown for 7 days into trophoblast organoids. Data are shown as mean ± S.D. (n=30). Statistical analysis was evaluated with an unpaired two-sample t-test. The viability was calculated using the ratio of total dead cell divided by the total number of nuclei. n.s. = not significant (p>0.05).

Viability: 97.7 ± 0.94% (mean±S.D.)

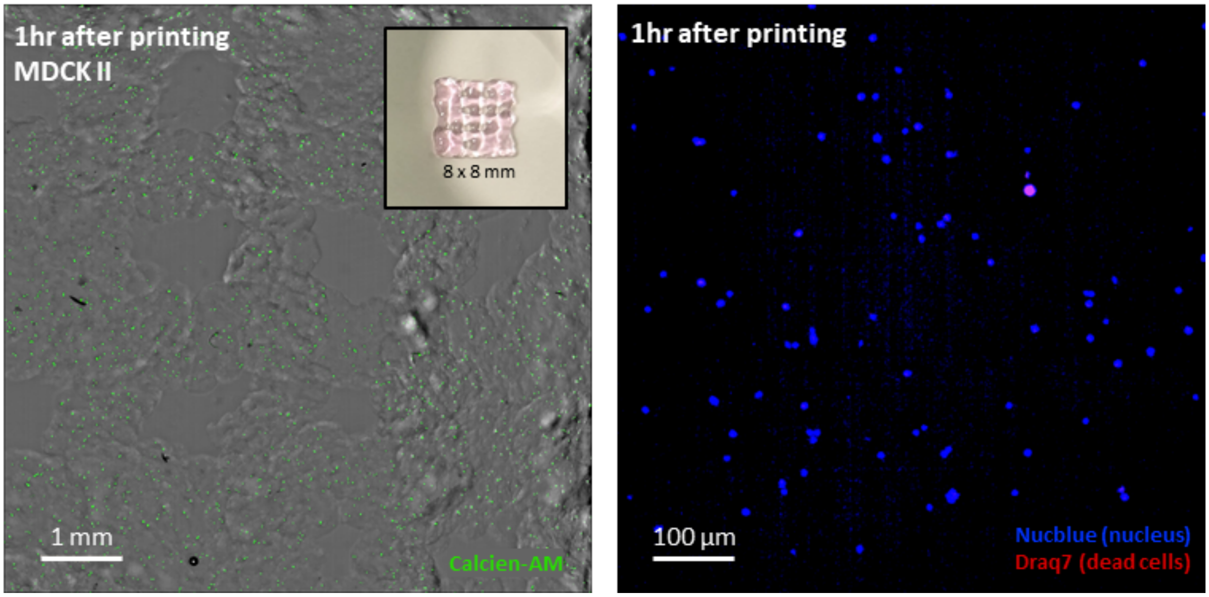

Figure S 14. Viability test with MDCK cells after extrusion printing. The cells were embedded in DyNAtrix (1% (w/v) $\boldsymbol{P_5^{RGD}}$ *+ CCL-64), and subsequently the cell-laden gels were extruded through a 1 mL syringe with a 30G nozzle on a BioScaffolder (GeSiM). 1 hour after printing, the cells were stained with Calcein-Am (live), Draq7 (dead), and NucBlue (nuclei). The viability was calculated using the ratio of total dead cell divided by the total number of nuclei in 4 structures that had been printed under identical conditions.*

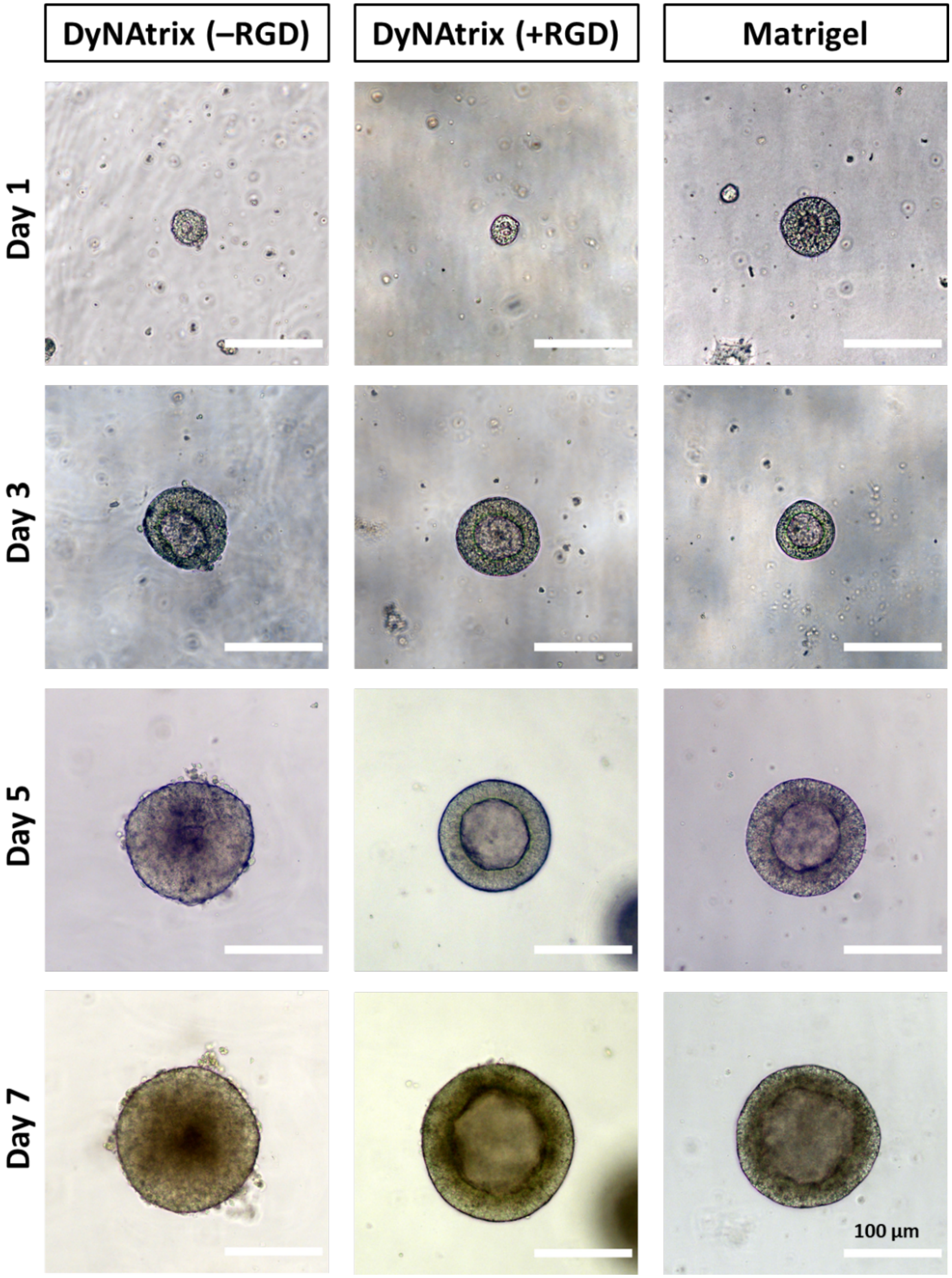

Figure S 15. Representative brightfield images of hiPSC cysts cultured in DyNAtrix [-RGD] (1% (w/v) $\boldsymbol{P_5}$ + CCL64) and DyNAtrix [+RGD] (1% (w/v) $\boldsymbol{P_5}^{RGD}$ + CCL-64) vs. Matrigel on day 1, 3, 5, and 7.

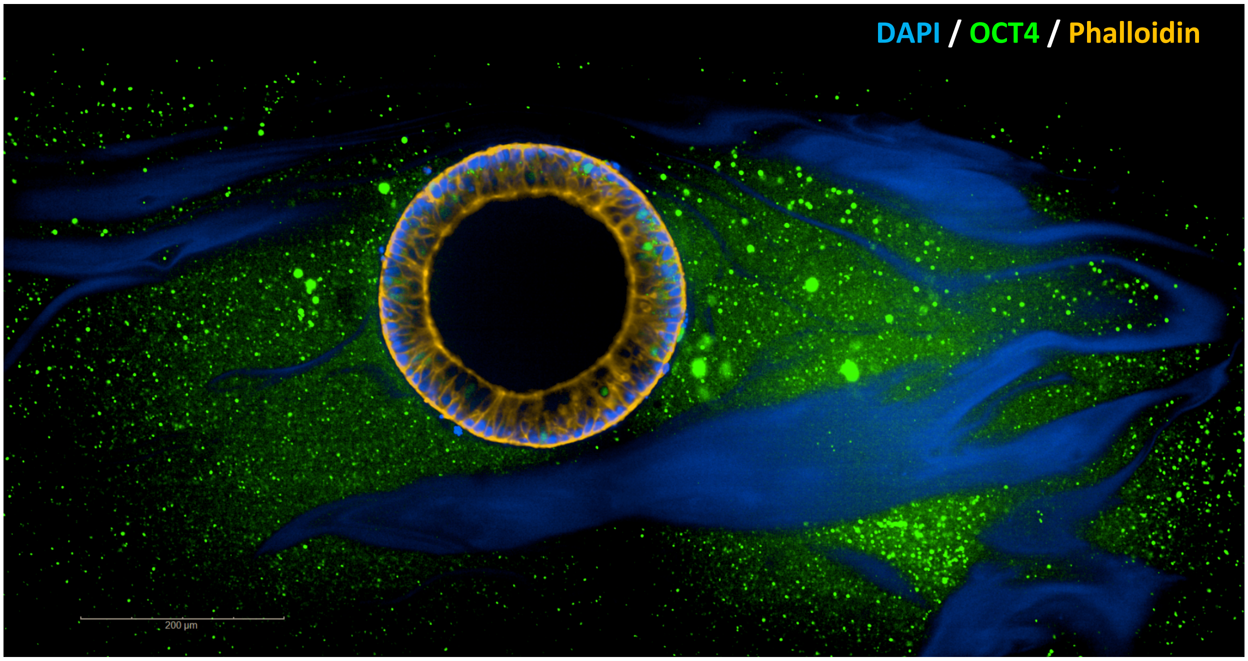

Figure S 16. Confocal microscope image of a hiPSC cyst stained inside DyNAtrix [+RGD] (1% (w/v) $\boldsymbol{P_{10}^{RGD}}$ + CCL-64) after culture for 7 days. The cells were stained with pluripotency marker OCT4 (green). F-actin was stained with Phalloidin (yellow). The cell nuclei were stained with DAPI (blue). The DAPI signal arising from extracellular DNA crosslinkers can be detectable, resulting in blue schlieren. The differences in DAPI signal are enhanced by background subtraction.

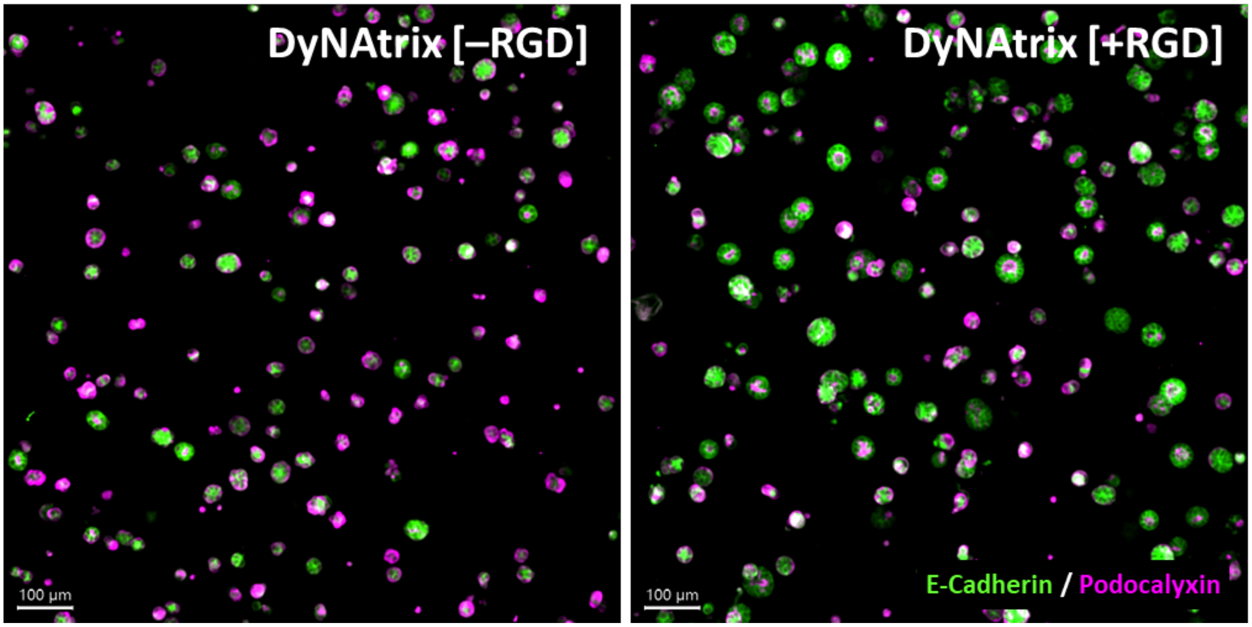

Figure S 17. Culture of MDCK cysts in DyNAtrix with 1% (w/v) $\boldsymbol{P_5}$ vs. $\boldsymbol{P_5^{RGD}}$ backbone crosslinked with CCL-64. A serum-free medium (UltraMDCK™) was used. Images were taken on day 5 of the culture. Scale bar = 100 µm.

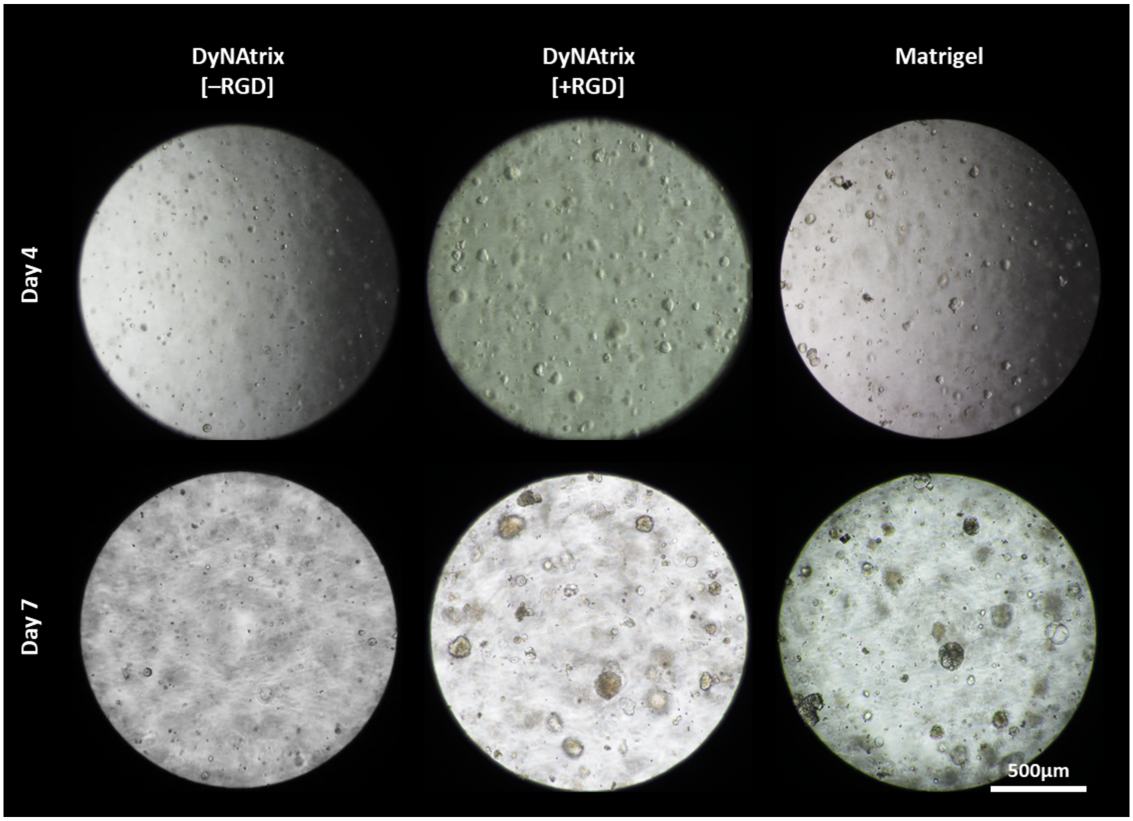

Figure S 18. Brightfield microscopy images of trophoblast organoids grown in DyNAtrix [+/–RGD] vs Matrigel (day 4 and 7).

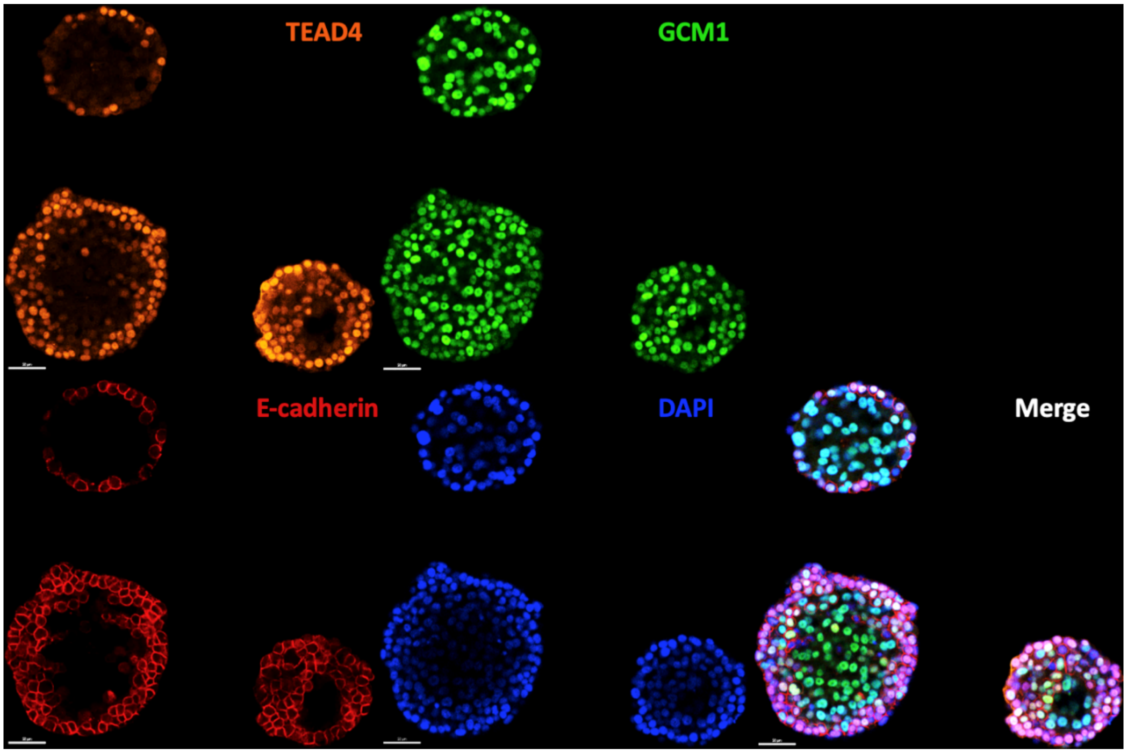

Figure S 19. Confocal images of trophoblast organoids grown in DyNAtrix (1% (w/v) $\mathbf{P}_{10}^{RGD}$ *+ CCL-64) on day 7 and stained for TEAD4, GCM1, E-Cadherin, and nuclei (DAPI). Scale bar: 50 µm.*

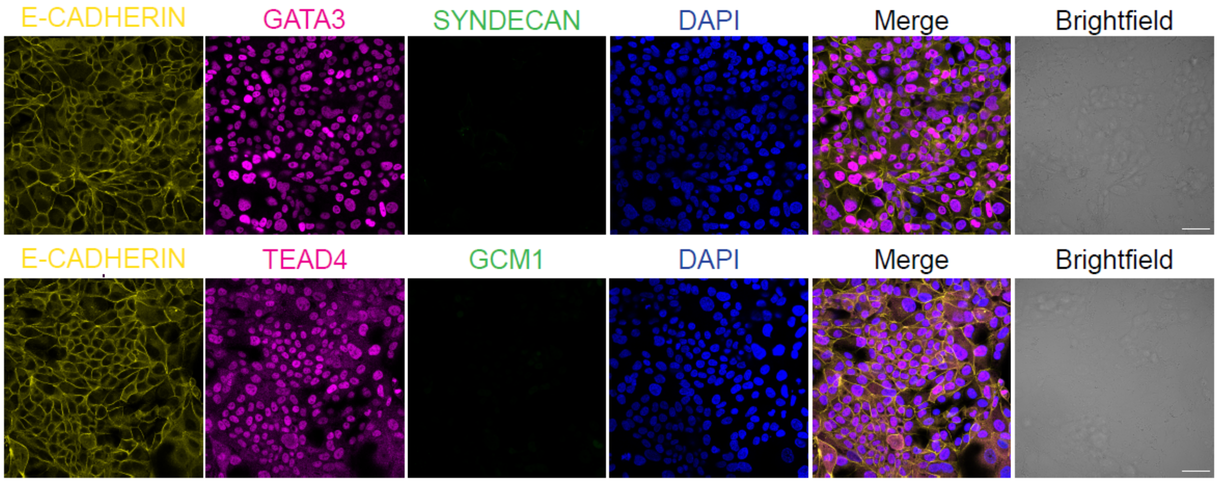

Figure S 20. Immunostaining of 2D cultured trophoblast cells. The cells were stained for GATA3, TEAD4, SYDECAN, GCM1, E-Cadherin, and DAPI (nuclei). The monolayer cells showed low expression of differentiation markers GCM1 and Syndecan, but strong expression of stemness markers TEAD4 and GATA3. Scale bar = 50 µm.

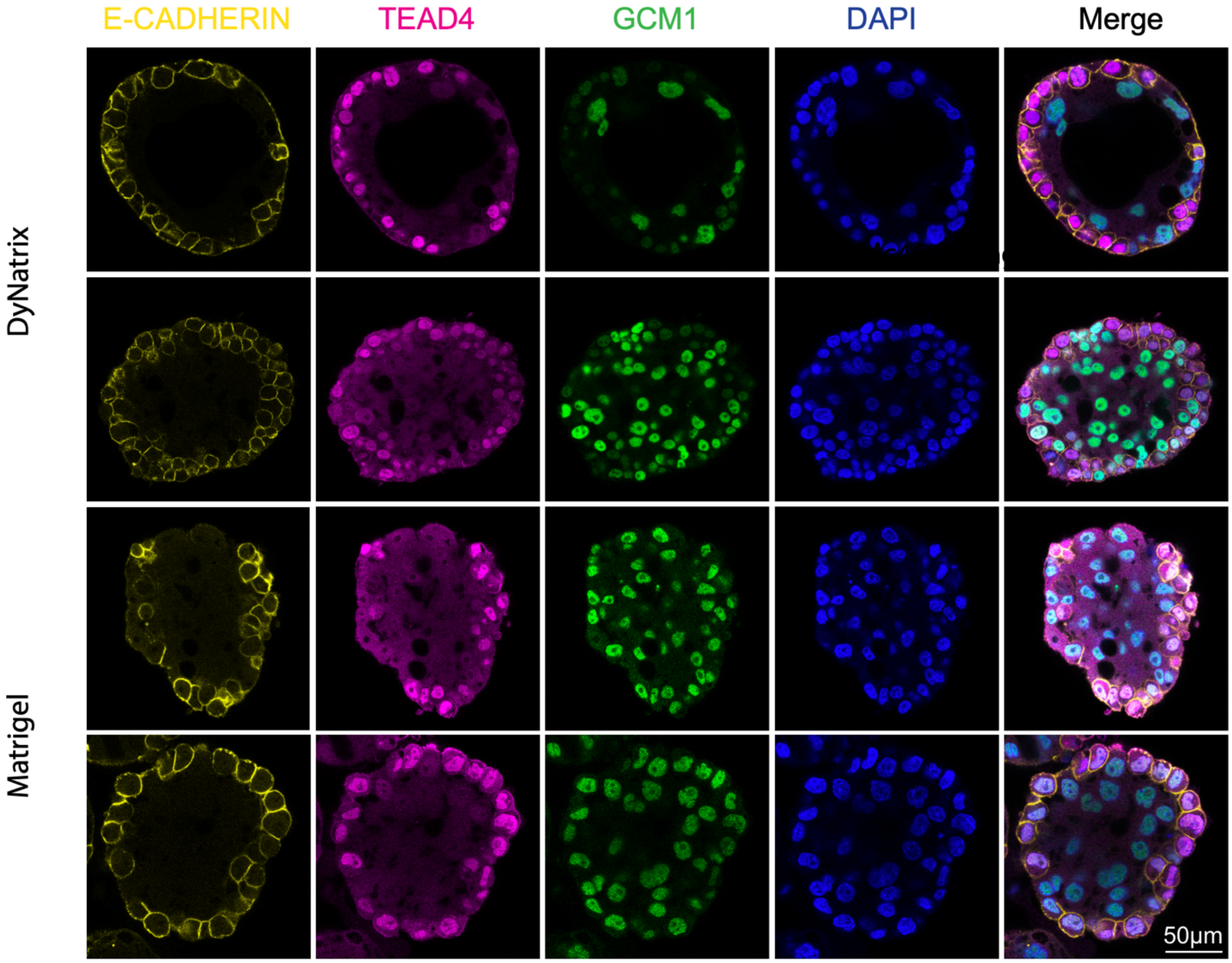

Figure S 21. Representative confocal images of several trophoblast organoids grown in DyNAtrix (1% (w/v) $\mathbf{P}_{10}^{RGD}$ *+ CCL-64) vs. Matrigel (day 7). Organoids were 100–200 µm in diameter and showed expression of E-Cadherin, TEAD4 and GCM1. Scale bar: 50 µm.*

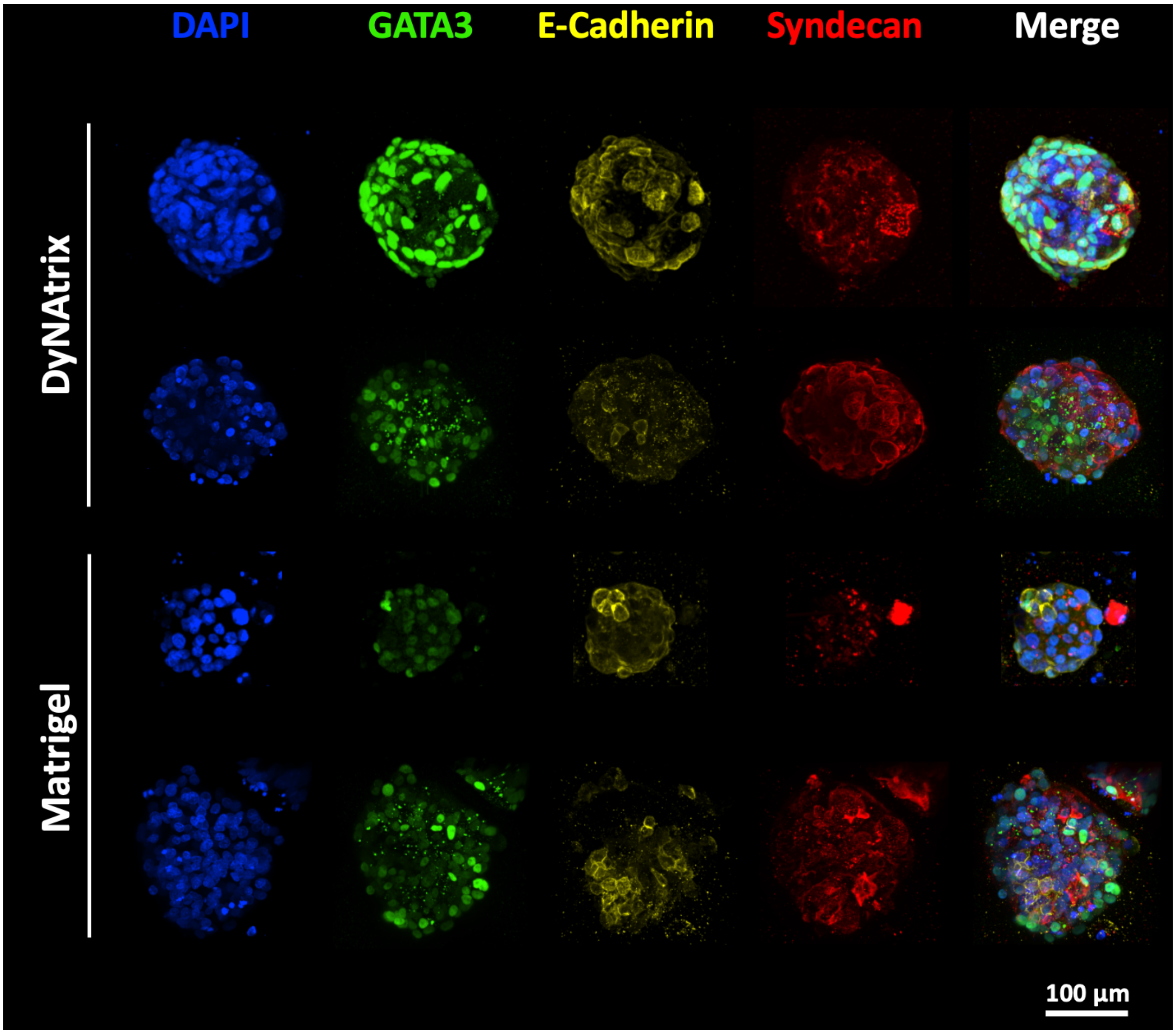

Figure S 22. Representative confocal images of several trophoblast organoids grown in DyNAtrix (1% (w/v) ***P_5^{RGD}*** *+ CCL-64) vs. Matrigel (day 7). Organoids were 100–200 µm in diameter and showed expression of GATA3, E-Cadherin and Syndecan.*

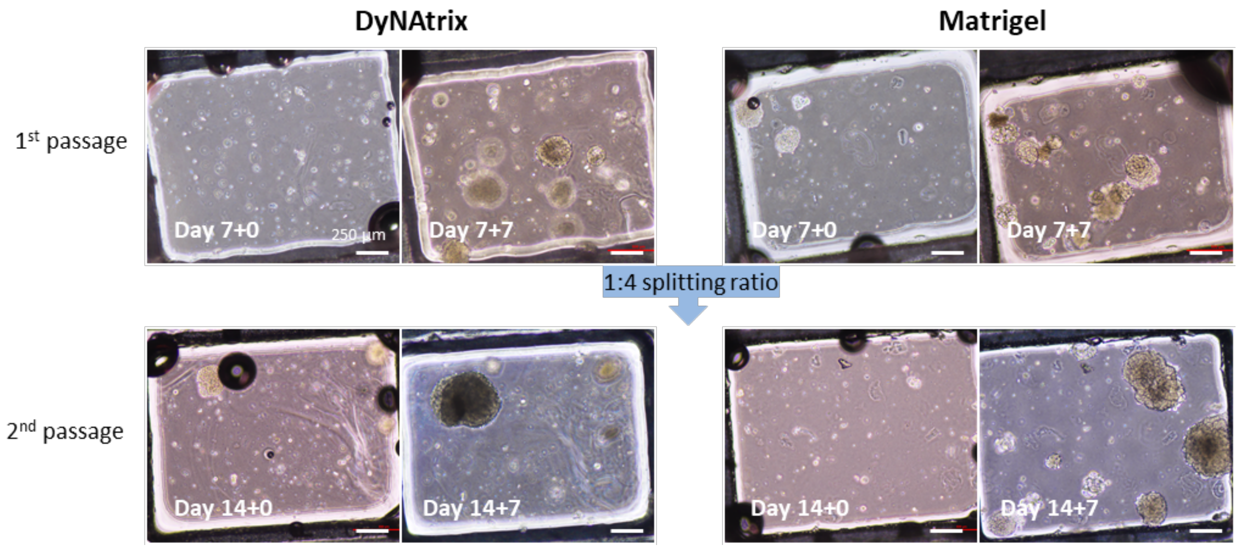

Figure S 23. Long-term trophoblast organoid culture for up to 21 days in DyNAtrix (1% (w/v) $\boldsymbol{P_{10}^{RGD}}$ *+ CCL-64) vs. Matrigel. Brightfield images show organoids that were passaged twice after an initial 3D culture for 7 days. The passaging workflow for DyNAtrix was adapted from a protocol that had been previously established for Matrigel.*[4] *Scale bars: 250 µm.*

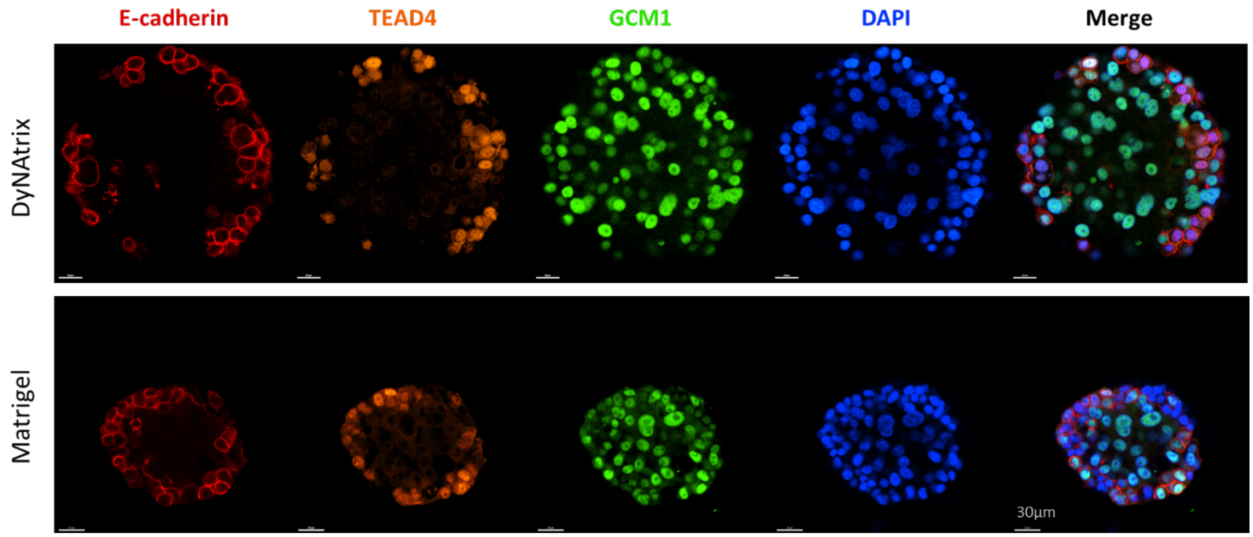

Figure S 24. Confocal images of 2nd passaged trophoblast organoids in DyNAtrix (1% (w/v) $\boldsymbol{P_{10}^{RGD}}$ *+ CCL-64) vs. Matrigel (day 14+7). The organoids showed expression of E-Cadherin, TEAD4 and GCM1, similar to the one before passaging. Scale bar: 30 µm.*

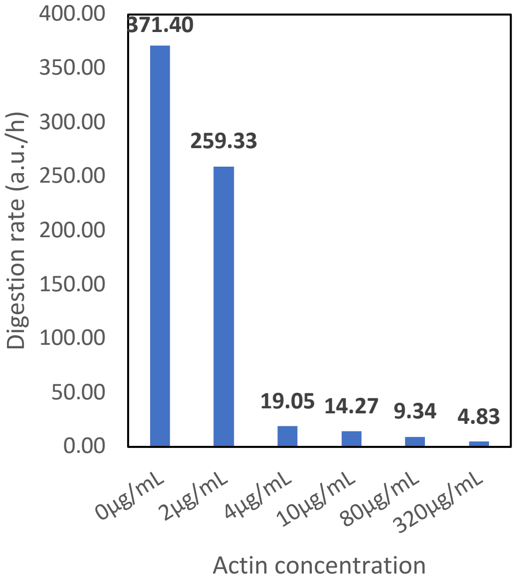

Figure S 25. The digestion rate of FRET-paired DNA in DNase-containing medium supplemented with different concentration of actin. The rate is determined by the slope of the linear region of the regression line in the beginning of the fluorescence curve (R^2 > 0.98).

4 Supplementary Tables

Table S 1. List of the DNA sequences used in this study. Red: adaptor site complementary to anchor strand; blue: overlap domain; magenta: ambiguous N base (A,T,C, or G). 5Acryd: Acrydite; IAbRQSp: Iowa black RQ-Sp; Cy5: cyanine 5; 6-FAM: 6-carboxyfluorescein.

#	Anchor strand	Length [nt]
1	/5Acryd/GACGGCTCATAAGGCTCTAATC	22

#	Single-splint crosslinker library	Length [nt]
2	GATTAGAGCCTTATGAGCCGTCGATTAGAGCCTTATGAGCCGTC	44

#	1-splint library (*CCL-1*)	Length [nt]
3a	TTAGTCAGTGTCCCGATTAGAGCCTTATGAGCCGTC	36
3b	GGGACACTGACTAAGATTAGAGCCTTATGAGCCGTC	36

#	4-splint library (*CCL-4*) (*SRC-14nt*)	Length [nt]
4a	TTAGTCANTGTCCCGATTAGAGCCTTATGAGCCGTC	36
4b	GGGACANTGACTAAGATTAGAGCCTTATGAGCCGTC	36

#	16-splint library (*CCL-16*)	Length [nt]
5a	TTAGTNAGTNTCCCGATTAGAGCCTTATGAGCCGTC	36
5b	GGGANACTNACTAAGATTAGAGCCTTATGAGCCGTC	36

#	64-splint library (*CCL-64*)	Length [nt]
6a	TTAGTNANTNTCCCGATTAGAGCCTTATGAGCCGTC	36
6b	GGGANANTNACTAAGATTAGAGCCTTATGAGCCGTC	36

#	256-splint library (*CCL-256*)	Length [nt]
7a	TTAGTNANTNTNCCGATTAGAGCCTTATGAGCCGTC	36
7b	GGNANANTNACTAAGATTAGAGCCTTATGAGCCGTC	36

#	18nt 4-splint library (*SRC-18nt*)	Length [nt]
8a	GTTTAGTCANTGTCCCGTGATTAGAGCCTTATGAGCCGTC	40
8b	ACGGGACANTGACTAAACGATTAGAGCCTTATGAGCCGTC	40

#	12nt 4-splint library (*SRC-12nt*)	Length [nt]
9a	TAGTCANTGTCCGATTAGAGCCTTATGAGCCGTC	34
9b	GGACANTGACTAGATTAGAGCCTTATGAGCCGTC	34

#	10nt 4-splint library (*SRC-10nt*)	Length [nt]
10a	AGTCANTGTCGATTAGAGCCTTATGAGCCGTC	32
10b	GACANTGACTGATTAGAGCCTTATGAGCCGTC	32

#	8nt 4-splint library (*SRC-8nt*)	Length [nt]
11a	GTCANTGTGATTAGAGCCTTATGAGCCGTC	30
11b	ACANTGACGATTAGAGCCTTATGAGCCGTC	30

#	6nt 4-splint library (*SRC-6nt*)	Length [nt]
12a	TCANTGGATTAGAGCCTTATGAGCCGTC	28
12b	CANTGAGATTAGAGCCTTATGAGCCGTC	28

#	FRET probe	Length [nt]
13a	/Cy5/CCGAGGACTGAGGGTTTTAGGAGTTGGTCTATAATCATGG	40
13b	AGACCAACTCCTAAAACCCTCAGTCCTCGG/IAbRQSp/	30

#	Heat-activated blocking strands (converts *CCL-4* into a *HAC*)	Length [nt]
14a	AAGACANTGACTGT	14
14b	ACAGTCANTGTCTT	14

#	Heat-activated blocking strands (converts *CCL-64* into a *HAC*)	Length [nt]
15a	AAGANANTNACTGT	14
15b	ACAGTNANTNTCTT	14

#	6-FAM 5'-end modified strand	Length [nt]
16	/56-FAM/AAGAGTACAGTCCAGATTAGAGCCTTATGAGCCGTC	36

#	Directly-grafted 64-splint library (*CCL-64*)	Length [nt]
17a	/5Acryd/TTAGTNANTNTCCC	14
17b	/5Acryd/GGGANANTNACTAA	14

Table S 2. AF4-LS characterization of DyNAtrix backbones.

	M_w *1 (kg/mol)	M_n *1 (kg/mol)	Đ (M_w/M_n)	R_g (nm)	R_h (nm)	R_g/R_h	ρ_{app} *2 (g/l)	V_h *3 (µm³)
$\mathbf{P}_1$	2,820	2,220	1.27	94	89	1.06	1.27	0.0029
$\mathbf{P}_5$	3,343	2,907	1.15	96	99	0.97	1.29	0.0041
$\mathbf{P}_5^{\mathrm{RGD}}$	3,450	2,530	1.43	96	71	1.38	3.38	0.0016
$\mathbf{P}_{10}$	2,280	2,140	1.06	91	60	1.53	3.99	0.0009
$\mathbf{P}_{10}^{\mathrm{RGD}}$	2,560	2,440	1.05	87	55	1.58	5.94	0.0007

M_W = mass average molecular weight; M_n = number average molecular weight; Đ = polydispersity index; R_g = radius of gyration; R_h = hydrodynamic radius; $\rho = \rho_{app}$ = apparent density; V_h = apparent volume

*1 dn/dc = 0.170 mL/g; *2 calculated from M_n and R_h; *3 calculated from R_h

Table S 3. Batch-to-batch *variation: comparison of the mass average polymer molecular weight, M_W, of three different synthesis products of* $\boldsymbol{P}_5$ *and three different synthesis products of* $\boldsymbol{P}_5^{RGD}$.

	M_w (kg/mol)	
	$\mathbf{P}_5$	$\mathbf{P}_5^{\mathrm{RGD}}$
Synthesis 1	3,360	2,960
Synthesis 2	3,950	3,680
Synthesis 3	2,720	3,710
**Average**	**3,343**	**3,450**
± Std. Dev.	615	347

Table S 4. Cost calculation of DyNAtrix.

Materials/ Reagents	Unit	Unit cost	Cost per mL of 1% gel
Anchor strand	1 nmol	€ 0.06	€ 5.72
Fmoc-L-Lys(Acryloyl)-OH	1 mg	€ 0.17	€ 1.38
DNA Crosslinkers	1 nmol	€ 0.08	€ 6.00
Heat-activated blocking strands	1 nmol	€ 0.08	€ 12.00
**DyNAtrix [+RGD]**			**€ 25.10**
Actin (optional)	1 µg	€ 0.10	€ 39.64
**DyNAtrix [+RGD] [+ actin]**			**€ 64.74**

5 Supplementary References

1. Schindelin, J. *et al.* Fiji: an open-source platform for biological-image analysis. *Nat Methods* **9**, 676–682 (2012).

2. Zhang, H. *et al.* Identification of distinct nanoparticles and subsets of extracellular vesicles by asymmetric flow field-flow fractionation. *Nat Cell Biol* **20**, 332–343 (2018).

3. Okae, H. *et al.* Derivation of Human Trophoblast Stem Cells. *Cell Stem Cell* **22**, 50-63.e6 (2018).

4. Sheridan, M. A. *et al.* Establishment and differentiation of long-term trophoblast organoid cultures from the human placenta. *Nat Protoc* **15**, 3441–3463 (2020).

5. Flory, P. J. *Principles of polymer chemistry*. (Ithaca : Cornell University Press, 1953., 1953).

6. Yoshihito Osada *et al. Gels Handbook, Four-Volume Set*. *Gels Handbook* (Academic Press, 2001). doi:https://doi.org/10.1016/B978-012394690-4/50142-0.

7. Aharoni, S. Intramolecular crosslinking. *Die Angewandte Makromolekulare Chemie* **62**, (1977).

8. Lin, D. C., Yurke, B. & Langrana, N. A. Mechanical Properties of a Reversible, DNA-Crosslinked Polyacrylamide Hydrogel . *J Biomech Eng* **126**, 104–110 (2004).

9. Akintayo, C. O., Creusen, G., Straub, P. & Walther, A. Tunable and Large-Scale Model Network StarPEG-DNA Hydrogels. *Macromolecules* **54**, 7125–7133 (2021).

10. Straube, E. Scaling concepts in polymer physics. Von P. G. DE GENNES. Ithaca/London: Cornell University Press 1980, *Acta Polymerica* **32**, 290 (1981).

11. Zadeh, J. N. *et al.* NUPACK: Analysis and design of nucleic acid systems. *J Comput Chem* **32**, 170–173 (2011).
