# Supplementary material for: Dynamic matrices with DNA-encoded viscoelasticity for cell and organoid culture
Source: Nat Nanotechnol. 2023 Aug 7;18(12):1463–73. doi: 10.1038/s41565-023-01483-3 (PMC10716043; doi:10.1038/s41565-023-01483-3)
Supplement: Supplementary file 2 — Reporting Summary [file 41565_2023_1483_MOESM2_ESM.pdf]

nature portfolio

Corresponding author(s): Elisha Krieg

Last updated by author(s): 29.06.2023

Reporting Summary

Nature Portfolio wishes to improve the reproducibility of the work that we publish. This form provides structure for consistency and transparency in reporting. For further information on Nature Portfolio policies, see our [Editorial Policies] and the [Editorial Policy Checklist].

Statistics

For all statistical analyses, confirm that the following items are present in the figure legend, table legend, main text, or Methods section.

n/a	Confirmed	
☐	☒	The exact sample size (*n*) for each experimental group/condition, given as a discrete number and unit of measurement
☐	☒	A statement on whether measurements were taken from distinct samples or whether the same sample was measured repeatedly
☐	☒	The statistical test(s) used AND whether they are one- or two-sided<br>*Only common tests should be described solely by name; describe more complex techniques in the Methods section.*
☒	☐	A description of all covariates tested
☒	☐	A description of any assumptions or corrections, such as tests of normality and adjustment for multiple comparisons
☐	☒	A full description of the statistical parameters including central tendency (e.g. means) or other basic estimates (e.g. regression coefficient) AND variation (e.g. standard deviation) or associated estimates of uncertainty (e.g. confidence intervals)
☐	☒	For null hypothesis testing, the test statistic (e.g. *F*, *t*, *r*) with confidence intervals, effect sizes, degrees of freedom and *P* value noted<br>*Give P values as exact values whenever suitable.*
☒	☐	For Bayesian analysis, information on the choice of priors and Markov chain Monte Carlo settings
☒	☐	For hierarchical and complex designs, identification of the appropriate level for tests and full reporting of outcomes
☒	☐	Estimates of effect sizes (e.g. Cohen's *d*, Pearson's *r*), indicating how they were calculated

Our web collection on statistics for biologists contains articles on many of the points above.

Software and code

Policy information about availability of computer code

Data collection	NIS-Elements (Nikon) was used to acquire bright-field images from Nikon Eclipse Ts2 inverted microscope. Fusion (Oxford Instrument) was used to acquire images from Andor Dragonfly Confocal microscope. Harmony (PerkinElmer) was used to acquire images on Opera Phenix Confocal microscope. GeSiM Robotics (GeSiM) was used to configure and control the printing process. RheoCompass (Anton Paar) was used to control the MCR301 rheometer. NUPACK (Caltech) was used to analyze and design the nuclei acid sequences.
Data analysis	Image processing was performed using Fiji-ImageJ and IMARIS (Oxford Instrument). Statistical analysis were performed using GraphPad Prism 9.5.0 and Origin 2022. qPCR data were visualized using CFX Maestro (Bio-Rad).

For manuscripts utilizing custom algorithms or software that are central to the research but not yet described in published literature, software must be made available to editors and reviewers. We strongly encourage code deposition in a community repository (e.g. GitHub). See the Nature Portfolio guidelines for submitting code & software for further information.

Data

Policy information about availability of data

All manuscripts must include a data availability statement. This statement should provide the following information, where applicable:

- Accession codes, unique identifiers, or web links for publicly available datasets
- A description of any restrictions on data availability
- For clinical datasets or third party data, please ensure that the statement adheres to our policy

The datasets supporting this study are included within the article and on Figshare (https://figshare.com/projects/Dynamic_matrices_with_DNA-encoded_viscoelasticity_for_cell_and_organoid_culture/168281)

Research involving human participants, their data, or biological material

Policy information about studies with human participants or human data. See also policy information about sex, gender (identity/presentation), and sexual orientation and race, ethnicity and racism.

Reporting on sex and gender	This information was not collected.
Reporting on race, ethnicity, or other socially relevant groupings	This information was not collected.
Population characteristics	The human whole blood was obtained from two voluntary ABO-matched donors who had not used any medicine in the past ten days.
Recruitment	The participants were selected when their blood types won't trigger instant coagulation to minimize the impact of natural biological reaction.
Ethics oversight	The donors' informed consent was obtained. The study involving human whole blood was covered by the ethic vote EK-BR-24/18-1 of the Sächsische Landesärztekammer.

Note that full information on the approval of the study protocol must also be provided in the manuscript.

Field-specific reporting

Please select the one below that is the best fit for your research. If you are not sure, read the appropriate sections before making your selection.

☒ Life sciences ☐ Behavioural & social sciences ☐ Ecological, evolutionary & environmental sciences

For a reference copy of the document with all sections, see nature.com/documents/nr-reporting-summary-flat.pdf

Life sciences study design

All studies must disclose on these points even when the disclosure is negative.

Sample size	No sample size calculation, allocation of samples to different groups and randomization was performed.
Data exclusions	No data acquired for analysis were excluded.
Replication	Cell culture conditions were typically tested with a minimum of three biological replicates.
Randomization	Cell clusters were randomly allocated into the experimental groups.
Blinding	Investigators were not blinded with respect to the identities of the samples. However, all experimental and control samples were collected at the same time under the same condition, and data analysis was carried out with the same software.

Reporting for specific materials, systems and methods

We require information from authors about some types of materials, experimental systems and methods used in many studies. Here, indicate whether each material, system or method listed is relevant to your study. If you are not sure if a list item applies to your research, read the appropriate section before selecting a response.

Materials & experimental systems

n/a	Involved in the study	
☐	☒	Antibodies
☐	☒	Eukaryotic cell lines
☒	☐	Palaeontology and archaeology
☒	☐	Animals and other organisms
☒	☐	Clinical data
☒	☐	Dual use research of concern
☒	☐	Plants

Methods

n/a	Involved in the study	
☒	☐	ChIP-seq
☒	☐	Flow cytometry
☒	☐	MRI-based neuroimaging

Antibodies

Antibodies used

The following primary antibodies were used for immunofluorescent staining:
Oct3/4 (BD Biosciences, Cat. # 611202, 1: 300)
E-cadherin (Invitrogen, Cat. # 13-1900, 1:200)
Syndecan (Sigma, Cat. # HPA006185, 1:200)
GATA3 (R&D, Cat. # AF2605, 1:200)
ENDOU (Sigma, Cat. # HPA012388, 1:200)
GCM1 (Atlas, Cat. # HPA011343, 1: 200)
TEAD4 (abcam, Cat. # ab58310, 1: 200)

Validation

All antibodies were verified by the supplier. Validation statements are available on the manufacturers' websites:
Oct3/4 has been validated for immunofluorescence and mentioned species reactivity with mouse and human. (https://www.bdbiosciences.com/en-de/products/reagents/microscopy-imaging-reagents/immunofluorescence-reagents/purified-mouse-anti-oct3-4.611202)
E-cadherin has been validated for immunofluorescence and mentioned species reactivity with mouse. (https://www.thermofisher.com/antibody/product/E-cadherin-Antibody-clone-ECCD-2-Monoclonal/13-1900)
Syndecan has been validated for immunofluorescence and mentioned species reactivity with human. (https://www.sigmaaldrich.com/DE/en/product/sigma/hpa006185)
GATA3 has been validated for immunofluorescence and mentioned species reactivity with human. (https://www.rndsystems.com/products/human-gata-3-antibody_af2605)
ENDOU has been validated for immunofluorescence and mentioned species reactivity with human. (https://www.sigmaaldrich.com/DE/en/product/sigma/hpa012388)
GCM1 has been validated for western blot and mentioned species reactivity with human. (https://www.atlasantibodies.com/products/antibodies/primary-antibodies/triple-a-polyclonals/gcm1-antibody-hpa011343/)
TEAD4 has been validated for western blot and mentioned species reactivity with human. (https://www.abcam.com/products/primary-antibodies/tead4-antibody-5h3-ab58310.html)

Eukaryotic cell lines

Policy information about cell lines and Sex and Gender in Research

Cell line source(s)

Human MSCs were isolated from healthy female/male donors (aged 26–37) by the Medical Faculty at the University Hospital Dresden. hiPSCs were generated from MACS-sorted CD34+ cells from the peripheral blood of a healthy donor (aged 20-24). The CT27 patient-derived trophoblast stem cell line used in this study had been derived from placental cytotrophoblast cells and was obtained from the RIKEN stem cell bank (RCB4936:CT27).

Authentication

MSC, hiPSC, and trophoblast stem cell were authenticated at the time of purchase.

Mycoplasma contamination

Mycoplasma infection was negative for all the cell lines in this study

Commonly misidentified lines
(See ICLAC register)

The cell lines used in this study are NOT present in the ICLAC register.
