# Supplementary figures and images for: Dynamic matrices with DNA-encoded viscoelasticity for cell and organoid culture
Source: Nat Nanotechnol. 2023 Aug 7;18(12):1463–73. doi: 10.1038/s41565-023-01483-3 (PMC10716043; doi:10.1038/s41565-023-01483-3)

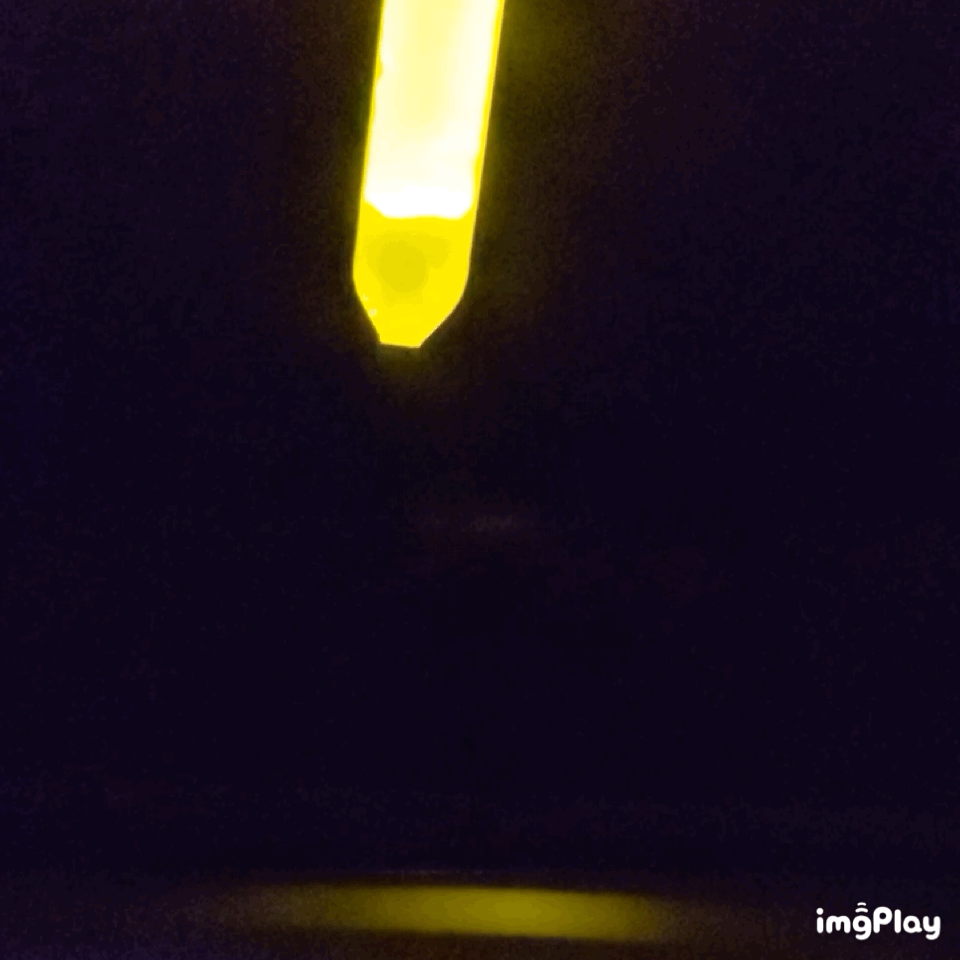

Supplement: Supplementary file 4 — Extrusion of DyNAtrix from a nozzle. [file 41565_2023_1483_MOESM4_ESM.gif]
